# Computational DFT data related to the redox behaviour of tris(β-diketonato)ruthenium(III) compounds

**DOI:** 10.1016/j.dib.2020.105617

**Published:** 2020-04-25

**Authors:** Jeanet Conradie

**Affiliations:** Department of Chemistry, PO Box 339, University of the Free State, Bloemfontein, 9300, South Africa

**Keywords:** Ruthenium, Acac, DFT, Relationships, HOMO, LUMO, Oxidation, Reduction

## Abstract

The data presented in this paper are related to the research article titled “*Redox Behaviour of [Ru(β-diketonato)_3_] Compounds*” [1]. This paper presents structural and energy data obtained from the density functional theory (DFT) computations. The energy data is related to experimentally obtained redox potential values. Various relationships are presented for the Ru^III/II^ and Ru^III/IV^ redox couples, involving both their experimental redox data as well as DFT calculated data, such as frontier orbital energies (*E*_HOMO_ and *E*_LUMO_) and calculated Mulliken electronegativity values.

Specifications Table

**Table:** 

Subject	Chemistry
Specific subject area	Computational chemistry
Type of data	Table Graph Figure
How data were acquired	Electronic structure calculations, using the Amsterdam Density Functional (ADF) 2018 and Gaussian 16 programmes.
Data format	Raw Analyzed
Parameters for data collection	Input coordinates were constructed manually, using ChemCraft
Description of data collection	Computational DFT data was obtained with the ADF 2018 and Gaussian 16 programmes on the High Performance Computing facility of the University of the Free State
Data source location	Department of Chemistry, University of the Free State, Nelson Mandela Street, Bloemfontein, South Africa
Data accessibility	Data is included with article and in the supplementary file
Related research article	J. Conradie, Redox Behaviour of [Ru(β-diketonato)_3_] Compounds. Electrochim. Acta. 337 (2020) 135801. https://doi:10.1016/j.electacta.2020.135801.

## Value of the Data

• Density functional theory (DFT) calculated optimized xyz-data (coordinates) for a series of 14 tris(β-diketonato)ruthenium(III) compounds are provided
• DFT optimized geometrical data (coordinates) can be used to visualize the DFT calculated structures of a series of 14 tris(β-diketonato)ruthenium(III) compounds
• This data provides highest occupied molecular orbital (HOMO) and lowest unoccupied molecular orbital (LUMO) energies (E_HOMO_ and E_LUMO_) of different tris(β-diketonato)ruthenium(III) compounds
• Relationships between experimental redox data and DFT calculated frontier orbital energies and calculated Mulliken electronegativity (χ) for tris(β-diketonato)ruthenium(III) compounds containing different electron donating and electron withdrawing substituents, obtained by different DFT methods, all produced similar R^2^ values
• E_HOMO_, E_LUMO_ and χ_calc_ data obtained by the different DFT methods show the same trend, namely [Ru(β-diketonato)_3_] compounds containing electron withdrawing substituents on the β-diketonato ligand have lower E_HOMO_ and E_LUMO_, and higher χ_calc_ values than [Ru(β-diketonato)_3_] compounds containing electron donating substituents on the β-diketonato ligand
• Electronic energy data of different spin states of neutral, oxidized and reduced tris(acetylacetonato)ruthenium(III) provide the lowest energy spin state of the neutral, oxidized and reduced tris(acetylacetonato)ruthenium(III)
• Linear relationships obtained from this data enable further prediction of the properties of novel complexes prior to synthesis, to be confirmed by laboratory tests

## 1 Data Description

**Fig. 1:**
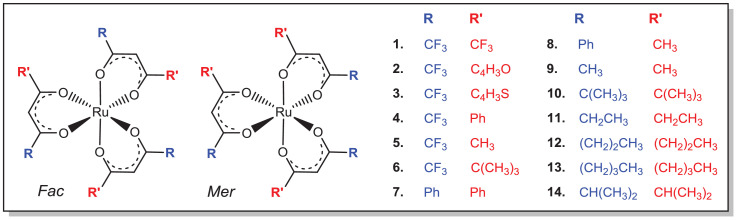
Structure of the fourteen [Ru(β-diketonato)_3_] compounds 1 – 14.

This data article provides data related to Ru(III) compounds **1** – **14** (Fig. 1). A summary of the Hammett meta-substituent sigma constants, σ_R_ [2], of both the R and Rꞌ substituents on the β-diketonato ligand of the [Ru(β-diketonato)_3_] compounds **1** – **14**, is provided in Table 1. The σ_R_ values provide an indication of the electron donating (smaller value) and electron withdrawing (larger value) property of the individual substituents R and Rꞌ on the β-diketonato ligand of the [Ru(β-diketonato)_3_] compounds **1** – **14**. On the other hand, the data of the sum (σ_R_ + σ_R'_) provides an indication of the electron donating (smaller value) and electron withdrawing (larger value) property of the β-diketonato ligand with its two substituents.

**Table 1:** Hammett meta-substituent sigma constants, σ_R_, of the individual R and Rꞌ groups [2] substituted on the β-diketonato ligand of the [Ru(β-diketonato)_3_] compounds 1 – 14, with the R and Rꞌ substituents as shown in Fig. 1. (σ_R_+ σ_Rꞌ_) gives the combined electronic effect of each ligand containing two substituents.

Compound no	R	R'	σ_R_	σ_R'_	(σ_R_ + σ_R'_)
1	CF_3_	CF_3_	0.43	0.43	0.86
2	CF_3_	C_4_H_3_O	0.43	0.06	0.49
3	CF_3_	C_4_H_3_S	0.43	0.09	0.52
4	CF_3_	Ph	0.43	0.06	0.49
5	CF_3_	CH_3_	0.43	-0.069	0.36
6	CF_3_	C(CH_3_)_3_	0.43	-0.1	0.33
7	Ph	Ph	0.06	0.06	0.12
8	CH_3_	Ph	-0.069	0.06	-0.01
9	CH_3_	CH_3_	-0.069	-0.069	-0.14
10	C(CH_3_)_3_	C(CH_3_)_3_	-0.10	-0.10	-0.20
11	Et	Et	-0.07	-0.07	-0.14
12	Pr	Pr	-0.06	-0.06	-0.12
13	Bu	Bu	-0.08	-0.08	-0.16
14	*i*Pr	*i*Pr	-0.04	-0.04	-0.08

Ru(III) compounds **1** – **14** (Fig. 1) were optimized by different density functional theory (DFT) methods in the solvent phase (CH_3_CN). Table 2 and Table 3 lists the DFT solvent phase (CH_3_CN) computed data, namely the highest occupied molecular orbital (HOMO) and lowest unoccupied molecular orbital (LUMO) energies (E_HOMO_ and E_LUMO_) and Mulliken electronegativity (χ_calc_, a measure of the tendency of an atom or molecule to attract electrons [3]) of the series of tris(β-diketonato)ruthenium(III) compounds **1** – **14** (Fig. 1). Experimental electrochemical data (potential *E vs* Fc/Fc^+^) of compounds **1** – **14**, obtained from literature [4,5], are also given in Table 2. Different E_HOMO_, E_LUMO_ and χ_calc_ values are obtained by the different DFT methods, though all methods show the same trend, namely [Ru(β-diketonato)_3_] compounds containing electron withdrawing substituents on the β-diketonato ligand (e.g. complexes **1** – **6** containing a CF_3_ group) have lower E_HOMO_ and E_LUMO_, and higher χ_calc_ values than [Ru(β-diketonato)_3_] compounds containing electron donating substituents on the β-diketonato ligand (e.g. complexes **9** – **14**), see Table 2 and Table 3.

**Table 2:** DFT calculated data from this data article, as well as experimental electrochemical data (*E vs* Fc/Fc^+^) obtained from literature [4,5], of the [Ru(β-diketonato)_3_] compounds 1 – 14. Where β-diketonato ligand = (RCOCHCORꞌ)^—^ with the R and Rꞌ substituents as shown in Fig. 1. DFT data was computed using two different gga functionals PW91 and OLYP.

	R	R'	*E* (Ru^III/II^)^a^	*E* (Ru^III/IV^)^a^	PW91/STO-TZ2P			OLYP/STO-TZ2P		
					E_HOMO_ (eV)	E_LUMO_ (eV)	χ_calc_ (eV)^c^	E_HOMO_ (eV)	E_LUMO_ (eV)	χ_calc_ (eV)^b^
**1**	CF_3_	CF_3_	0.34		-5.856	-5.585	5.720	-5.573	-5.337	5.455
**2**	CF_3_	C_4_H_3_O	-0.34	1.20	-5.067	-4.744	4.906	-4.793	-4.522	4.657
**3**	CF_3_	C_4_H_3_S	-0.35	1.19	-5.014	-4.753	4.883	-4.766	-4.486	4.626
**4**	CF_3_	Ph	-0.35	1.26	-5.134	-4.838	4.986	-4.865	-4.606	4.736
**5**	CF_3_	CH_3_	-0.47	1.29	-5.132	-4.812	4.972	-4.869	-4.596	4.733
**6**	CF_3_	C(CH_3_)_3_	-0.55	1.30	-5.065	-4.777	4.921	-4.782	-4.513	4.648
**7**	Ph	Ph	-0.90	0.66	-4.622	-4.294	4.458	-4.339	-4.070	4.205
**8**	CH_3_	Ph	-1.04	0.64	-4.529	-4.193	4.361	-4.260	-3.969	4.114
**9**	CH_3_	CH_3_	-1.16	0.61	-4.437	-4.137	4.287	-4.176	-3.911	4.044
**10**	C(CH_3_)_3_	C(CH_3_)_3_	-1.46	0.44	-4.125	-3.843	3.984	-3.823	-3.581	3.702
**11**	Et	Et	-1.308	0.549	-4.335	-4.004	4.170	-4.056	-3.785	3.921
**12**	Pr	Pr	-1.324	0.547	-4.316	-4.001	4.158	-4.039	-3.768	3.903
**13**	Bu	Bu	-1.330	0.535	-4.307	-3.979	4.143	-4.024	-3.766	3.895
**14**	iPr	iPr	-1.392	0.509	-4.196	-3.930	4.063	-3.900	-3.682	3.791

a Experimental values for *E vs* Fc/Fc^+^ from references [4,5]. In order to convert to potential *vs* Fc/Fc^+^ for comparative reasons, the following values have been used: *E*°' (Fc/Fc^+^) = 0.66(5) V *vs* NHE in solvent [^n^(Bu_4_)N][PF_6_]/CH_3_CN [9]; Saturated calomel (SCE) = 0.2444 V *vs* NHE; Ag/Ag^+^ = 0.400 V *vs* SCE [10].

b χ = Electronegativity

**Table 3:** DFT calculated data from this data article, as well as experimental electrochemical data (*E vs* Fc/Fc^+^) obtained from literature [4,5], of the [Ru(β-diketonato)_3_] compounds 1 – 14. Where β-diketonato ligand = (RCOCHCORꞌ)^—^ with the R and Rꞌ substituents as shown in Fig. 1. DFT data was computed using two different hybrid functionals OPBE0 and B3LYP.

	R	R'	*E* (Ru^III/II^) a	*E* (Ru^III/IV^) a	OPBE0/STO-TZ2P			B3LYP/STO-TZ2P			B3LYP/STO-6-311G(d,p)/Lanl2dz		
					E_HOMO_ (eV)	E_LUMO_ (eV)	χ_calc_ (eV) ^c^	E_HOMO_ (eV)	E_LUMO_ (eV)	χ_calc_ (eV) ^c^	E_HOMO_ (eV)	E_LUMO_ (eV)	χ_calc_ (eV) ^c^
**1**	CF_3_	CF_3_	0.34		-7.183	-4.200	5.692	-6.938	-4.558	5.748	-7.507	-3.272	5.389
**2**	CF_3_	C_4_H_3_O	-0.34	1.20	-6.404	-3.408	4.906	-6.103	-3.725	4.914	-6.607	-2.768	4.688
**3**	CF_3_	C_4_H_3_S	-0.35	1.19	-6.393	-3.410	4.901	-6.074	-3.707	4.890	-6.635	-2.822	4.729
**4**	CF_3_	Ph	-0.35	1.26	-6.528	-3.482	5.005	-6.168	-3.765	4.967	-6.777	-2.758	4.767
**5**	CF_3_	CH_3_	-0.47	1.29	-6.519	-3.413	4.966	-6.211	-3.792	5.001	-6.815	-2.414	4.615
**6**	CF_3_	C(CH_3_)_3_	-0.55	1.30	-6.420	-3.341	4.881	-6.165	-3.732	4.949	-6.745	-2.353	4.549
**7**	Ph	Ph	-0.90	0.66	-5.987	-2.978	4.483	-5.620	-3.211	4.415	-6.229	-2.405	4.317
**8**	CH_3_	Ph	-1.04	0.64	-5.922	-2.863	4.393	-5.595	-3.132	4.364	-6.216	-2.137	4.177
**9**	CH_3_	CH_3_	-1.16	0.61	-5.863	-2.719	4.291	-5.215	-2.785	4.000	-6.166	-1.634	3.900
**10**	C(CH_3_)_3_	C(CH_3_)_3_	-1.46	0.44	-5.467	-2.403	3.935	-5.215	-2.785	4.000	-6.021	-1.474	3.748
**11**	Et	Et	-1.308	0.549	-5.722	-2.588	4.155	-5.435	-2.962	4.199	-6.151	-1.648	3.900
**12**	Pr	Pr	-1.324	0.547	-5.703	-2.577	4.140	-5.415	-2.938	4.177	-6.136	-1.633	3.884
**13**	Bu	Bu	-1.330	0.535	-5.681	-2.565	4.123	-5.415	-2.946	4.180	-6.130	-1.619	3.874
**14**	iPr	iPr	-1.392	0.509	-5.523	-2.470	3.996	-5.289	-2.836	4.062	-6.123	-1.591	3.857

a Experimental values for *E vs* Fc/Fc^+^ from references [4,5]. In order to convert to potential *vs* Fc/Fc^+^ for comparative reasons, the following values have been used: *E*°' (Fc/Fc^+^) = 0.66(5) V *vs* NHE in solvent [^n^(Bu_4_)N][PF_6_]/CH_3_CN [9]; Saturated calomel (SCE) = 0.2444 V *vs* NHE; Ag/Ag^+^ = 0.400 V *vs* SCE [10]. ^b^χ = Electronegativity

**Fig. 2:**
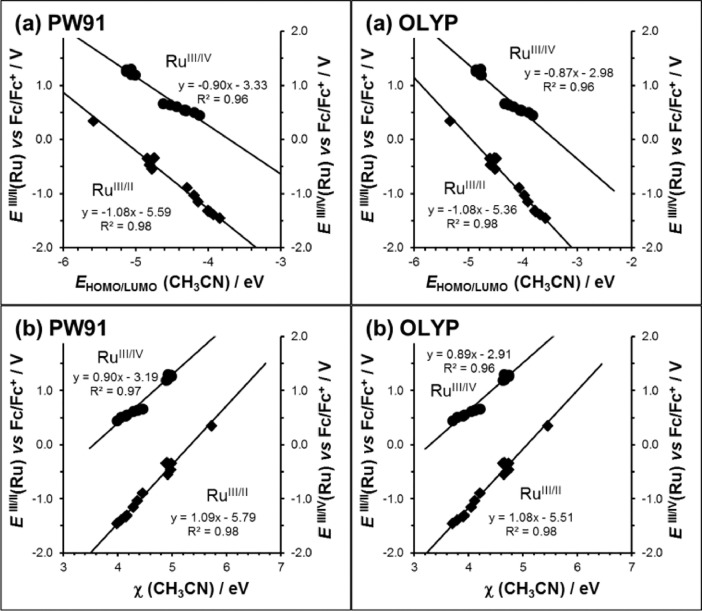
Relationships obtained between the experimental redox potential *E*°' (*vs* Fc/Fc^+^) of both the reduction (Ru^III/II^) and the oxidation (Ru^III/IV^) redox couples of the fourteen [Ru(β-diketonato)_3_] compounds 1 – 14 of this data article, with the DFT calculated data, namely (a) the LUMO (Ru^III/II^) and HOMO (Ru^III/IV^) energies *E*_HOMO/LUMO_, (b) calculated Mulliken electronegativity χ. All calculations were conducted in CH_3_CN as solvent, using the indicated gga functionals.

**Fig. 3:**
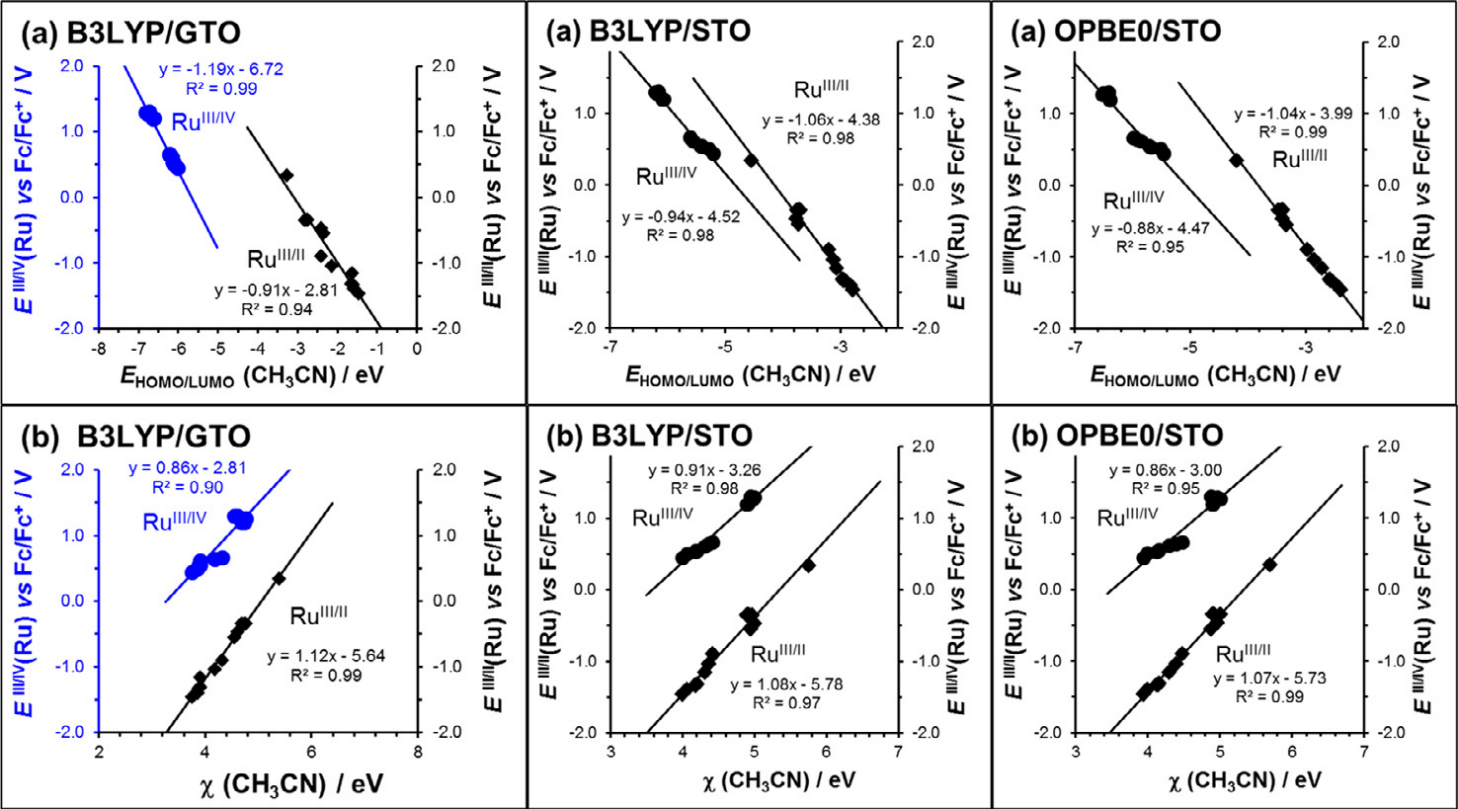
Relationships obtained between the experimental redox potential *E*°' (*vs* Fc/Fc^+^) of both the reduction (Ru^III/II^) and the oxidation (Ru^III/IV^) redox couples of the fourteen [Ru(β-diketonato)_3_] compounds 1 – 14 of this data article, with the DFT calculated data, namely (a) the LUMO (Ru^III/II^) and HOMO (Ru^III/IV^) energies *E*_HOMO/LUMO_ and (b) calculated Mulliken electronegativity χ. All calculations were conducted in CH_3_CN as solvent, using the indicated hybrid functionals.

The relationships between the experimental values of the reduction (Ru^III/II^) and oxidation (Ru^III/IV^) couples [4,5] and the solvent (CH_3_CN) phase calculated *E*_HOMO_ and *E*_LUMO_ energies and their χ_calc_ and ω_calc_ values, obtained via different DFT methods using generalized gradient approximations (gga) functionals, PW91/TZ2P and OLYP/TZ2P, are shown in Fig. 2 and obtained via different DFT methods using hybrid functionals, B3LYP/6-311G(d,p)/Lanl2dz, B3LYP/TZ2P and OPBE0/TZ2P, are shown in Fig. 3. The relationships obtained by these different solvent phase DFT methods, taking the experimental solvent (CH_3_CN) used for electrochemical experiments [4,5] into account in the calculations, all produced similar R^2^ values, comparable with the gas phase B3LYP/6-311G(d,p)/Lanl2dz calculated relationships obtained from reference [1]. The slopes of the experimental Ru^III/II^ and Ru^III/IV^ redox values *versus* the solvent (CH_3_CN) phase calculated *E*_HOMO_ and *E*_LUMO_ energies, are steeper than the corresponding gas phase calculated slope and also closer to nearing a gradient of -1.

Redox potentials and frontier orbital energies

Oxidation redox couple Ru^III/IV^:

**Table:** 

*E*°'(Ru^III/IV^) = -0.80 *E*_HOMO_(Ru^III^) – 4.00	R^2^ = 0.98	(gas phase B3LYP/6-311G(d,p)/Lanl2dz) [1]
*E*°'(Ru^III/IV^) = -1.19 *E*_HOMO_(Ru^III^) – 6.72	R^2^ = 0.99	(CH_3_CN phase B3LYP/6-311G(d,p)/Lanl2dz)
*E*°'(Ru^III/IV^) = -0.94 *E*_HOMO_(Ru^III^) – 4.52	R^2^ = 0.98	(CH_3_CN phase B3LYP/TZ2P)
*E*°'(Ru^III/IV^) = -0.88 *E*_HOMO_(Ru^III^) – 4.47	R^2^ = 0.95	(CH_3_CN phase OPBE0/TZ2P)
*E*°'(Ru^III/IV^) = -0.90 *E*_HOMO_(Ru^III^) – 3.33	R^2^ = 0.96	(CH_3_CN phase PW91/TZ2P)
*E*°'(Ru^III/IV^) = -0.87 *E*_HOMO_(Ru^III^) – 2.98	R^2^ = 0.96	(CH_3_CN phase OLYP/TZ2P)

Reduction redox couple Ru^III/II^:

**Table:** 

*E*°'(Ru^III/II^) = -0.72 *E*_LUMO_(Ru^III^) – 2.21	R^2^ = 0.98	(gas phase B3LYP/6-311G(d,p)/Lanl2dz) [1]
*E*°'(Ru^III/II^) = -0.91 *E*_LUMO_(Ru^III^) – 2.81	R^2^ = 0.94	(CH_3_CN phase B3LYP/6-311G(d,p)/Lanl2dz)
*E*°'(Ru^III/II^) = -1.06 *E*_LUMO_(Ru^III^) – 4.38	R^2^ = 0.98	(CH_3_CN phase B3LYP/TZ2P)
*E*°'(Ru^III/II^) = -1.04 *E*_LUMO_(Ru^III^) – 3.99	R^2^ = 0.99	(CH_3_CN phase OPBE0/TZ2P)
*E*°'(Ru^III/II^) = -1.08 *E*_LUMO_(Ru^III^) – 5.59	R^2^ = 0.98	(CH_3_CN phase PW91/TZ2P)
*E*°'(Ru^III/II^) = -1.08 *E*_LUMO_(Ru^III^) – 5.36	R^2^ = 0.98	(CH_3_CN phase OLYP/TZ2P)

HOMO (LUMO) energies are directly related to the absolute oxidation potential since the product of the HOMO (LUMO) energies (in eV) and the electron charge (-1) gives absolute oxidation potential in eV [6]. The nearer the slope of the graph of oxidation (reduction) potential *versus* HOMO (LUMO) energies is to -1, the more accurate the DFT method used to calculate the HOMO (LUMO) energies. The intercept of the graph should be equal to the absolute potential of reference used, namely the Fc^+^/Fc couple in acetonitrile for which benchmark values varies between +4.97 V (SMDB3LYP-D2/def2-QZVPPD//B3LYP/LanL2TZf/6-31G(d)) [7] and 4.988 V (G3(MP2)-RAD-Full-TZ using gas-phase energies and COSMO-RS solvation energies) [8]. In this study slopes of 0.7 – 1.2 and intercepts of 2.21 – 6.71 are obtained.

Redox potentials and global Mulliken electronegativity:

Oxidation redox couple Ru^III/IV^:

**Table:** 

*E*°'(Ru^III/IV^) = 0.67 χ_calc_ – 1.80	R^2^ = 0.96	(gas phase B3LYP/6-311G(d,p)/Lanl2dz) [1]
*E*°'(Ru^III/IV^) = 0.86 χ_calc_ – 2.81	R^2^ = 0.90	(CH_3_CN phase B3LYP/6-311G(d,p)/Lanl2dz)
*E*°'(Ru^III/IV^) = 0.91 χ_calc_ – 3.26	R^2^ = 0.98	(CH_3_CN phase B3LYP/TZ2P)
*E*°'(Ru^III/IV^) = 0.86 χ_calc_ – 3.00	R^2^ = 0.95	(CH_3_CN phase OPBE0/TZ2P)
*E*°'(Ru^III/IV^) = 0.90 χ_calc_ – 3.19	R^2^ = 0.97	(CH_3_CN phase PW91/TZ2P)
*E*°'(Ru^III/IV^) = 0.89 χ_calc_ – 2.91	R^2^ = 0.96	(CH_3_CN phase OLYP/TZ2P)

Reduction redox couple Ru^III/II^:

**Table:** 

*E*°'(Ru^III/II^) = 0.81 χ_calc_ – 4.09	R^2^ = 0.98	(gas phase B3LYP/6-311G(d,p)/Lanl2dz) [1]
*E*°'(Ru^III/II^) = 1.12 χ_calc_ – 5.64	R^2^ = 0.99	(CH_3_CN phase B3LYP/6-311G(d,p)/Lanl2dz)
*E*°'(Ru^III/II^) = 1.08 χ_calc_ – 5.78	R^2^ = 0.97	(CH_3_CN phase B3LYP/TZ2P)
*E*°'(Ru^III/II^) = 1.07 χ_calc_ – 5.93	R^2^ = 0.99	(CH_3_CN phase OPBE0/TZ2P)
*E*°'(Ru^III/II^) = 1.09 χ_calc_ – 5.79	R^2^ = 0.98	(CH_3_CN phase PW91/TZ2P)
*E*°'(Ru^III/II^) = 1.08 χ_calc_ – 5.51	R^2^ = 0.98	(CH_3_CN phase OLYP/TZ2P)

The energies relative to the ground state energy for the different possible spin states of the neutral, oxidized and reduced [Ru(acetylacetonato)_3_] compound **9** are provided in Table 4. The lowest energy value for each spin state showed that the neutral compound is low spin, S = ½ (doublet, one unpaired electron), in agreement with experiment [11]. The anion is diamagnetic with S = 0 (singlet), and the cation is paramagnetic with S = 1 (triplet, two unpaired electrons).

**Table 4:** DFT calculated relative energy (eV) data obtained from this data article, for the different possible spin states of the neutral, oxidized and reduced [Ru(acetylacetonato)_3_], complex 9. The lowest energy value for each of the neutral, oxidized and reduced states, is taken as 0.

	Spin	B3LYP	PW91
anion	0	0.00	0.00
	1	1.27	1.51
	2	-	2.41
neutral	1/2	0.00	0.00
	3/2	1.42	1.61
	5/3	2.15	3.84
cation	0	0.40	0.27
	1	0.00	0.00
	2	0.00	1.79

## 2 Experimental Design, Materials, and Methods

DFT calculations on all fourteen [Ru(β-diketonato)_3_] compounds were performed in the CH_3_CN solvent phase, using the following DFT methods:

(i) B3LYP/GTO-6-311G(d,p)/Lanl2dz: The hybrid functional B3LYP, which is composed of the Becke 88 exchange functional was applied in combination with the LYP correlation functional, as implemented in the Gaussian 16 package [12], applying the GTO (Gaussian type orbital) triple-ζ basis set 6-311G(d,p) for the lighter atoms (C, H, N, O, F) and the Lanl2dz (Los Alamos National Laboratory 2-double-ζ) basis set for the heavier Ru metal. The optimization is performed using Berny algorithm using GEDIIS [13] as implemented in the Gaussian 16 suite of programs [12]. The convergence is reached when the root mean square force, the maximum force, the root mean square displacement and the maximum displacement are within the threshold of 0.00030, 0.00045, 0.0012 and 0.0018 atomic units, respectively. The requested convergence on energy is 1.0D-6 atomic unit. The solvation model density (SMD) of the polarizable continuum model (PCM) was used, which also solved the non-homogeneous Poisson equation, by applying the integral equation formalism variant (IEF-PCM), as implemented in the Gaussian 16 package [12].
(ii) PW91/STO-TZ2P: Scalar-relativistic DFT using the gga PW91 (Perdew-Wang 1991) functional with the all-electron STO (Slater-Type Orbitals) triple ζ basis set with two polarization functions (TZ2P) was applied, as implemented in the ADF 2018 package [14]. The geometry optimizations procedure in ADF is based on a quasi Newton approach, with an approximate Hessian. The Hessian is updated in the process of optimization. By default delocalized coordinates are used. The default convergence criteria were used, namely 10^−3^ Hartree for the energy and 10^−3^ Hartree/Angstrom for the nuclear gradients. Solvent effects were taken into account for selected structures reported here, using the COSMO (Conductor like Screening Model) model of solvation, as implemented [15] in ADF. The type of cavity used was Esurf and the solvent used was CH_3_CN (*ε*_0_ = 37.5).
(iii) OLYP/STO-TZ2P: The gga OLYP functional was applied, with the TZ2P basis set and COSMO solvent model, as implemented in the ADF 2018 package [14].
(iv) OPBE0/STO-TZ2P: The hybrid OPBE0 functional was applied, with the TZ2P basis set and COSMO solvent model, as implemented in the ADF 2018 package [14].
(v) B3LYP/STO-TZ2P: The hybrid B3LYP functional was applied, with the TZ2P basis set and COSMO solvent model, as implemented in the ADF 2018 package [14].

The [Ru(β-diketonato)_3_] compounds were calculated as doublets (with S = ½) [11]. The input coordinates for the compounds were constructed using the program ChemCraft [16], and ChemCraft was also used to visualize the output files. The optimized coordinates, as well as an example input file, are provided in the supplementary information.

The DFT highest occupied molecular orbital (HOMO) and lowest unoccupied molecular orbital (LUMO) energies (*E*_HOMO_ and *E*_LUMO_) were obtained from the output file of the DFT computations. These energies were used to further calculate both the electron affinity (EA) and ionization potential (IP) of each of the fourteen compounds, according to Koopman's theorem [17,18]:

$$\text{IP}=-E_{\text{HOMO}}$$

and

$$\text{EA}=-E_{\text{LUMO}}$$

The Mulliken electronegativity (χ) [19] [20] was computed for each compound, by application of the following formulae:

χ=(IP+EA)/2

For the unsymmetrically substituted compounds **2** – **6** and **8** where R ≠ Rꞌ, an effective calculated energy (*E*_HOMO_ and *E*_LUMO_) was determined by using the ratio of the relative population of the *fac* and *mer* isomers (*n*_i_ or *n*_j_), as determined by the Boltzmann equation at T = 298.15 K:

$$\ln\frac{n_{j}}{n_{i}}=-\frac{\left(E_{j}-E_{i}\right)}{kT}$$

where *n*_i_ is the number of molecules with energy *E*_i_ (*fac* or *mer* in this case), with the Boltzmann's constant, k = 1.38066 × 10^23^ JK^−1^. *E*_i_ are provided in the supplementary information.

## Appendix Supplementary materials

Image, application 1

Image, application 2

Image, application 3
