# Supplementary material for: Computational DFT data related to the redox behaviour of tris(β-diketonato)ruthenium(III) compounds
Source: Data Brief. 2020 Apr 25;30:105617. doi: 10.1016/j.dib.2020.105617 (PMC7206207; doi:10.1016/j.dib.2020.105617)
Supplement: Supplementary file 1 [file mmc1.pdf]

Data Article

Computational DFT data related to the redox behaviour of tris(β-diketonato)ruthenium(III) compounds.

Jeanet Conradie

Department of Chemistry, PO Box 339, University of the Free State, Bloemfontein, 9300, South Africa

Corresponding author
conradj@ufs.ac.za

Supporting information

Table of Contents

Data Article 1
Electronic energy data 2
Example input files 2
(i) B3LYP/GTO-6-311G(d,p)/Lanl2dz (Gaussian 16) 2
(ii) PW91/STO-TZ2P (ADF 2018) 4
(iii) OLYP/STO-TZ2P (ADF 2018) 5
(iv) B3LYP/STO-TZ2P (ADF 2018) 7
(v) OPBE0/STO-TZ2P (ADF 2018) 9
Optimized Cartesian coordinates (Å) for PW91/TZ2P 11
1) [Ru($CF_3COCHCOCF_3$)$_3$] complex 1 11
2) [Ru($CF_3COCHCO(C_4H_3O)$)$_3$] *fac* complex 2 12
3) [Ru($CF_3COCHCO(C_4H_3O)$)$_3$] *mer* complex 2 13
4) [Ru($CF_3COCHCO(C_4H_3S)$)$_3$] *fac* complex 3 15
5) [Ru($CF_3COCHCO(C_4H_3S)$)$_3$] *mer* complex 3 16
6) [Ru($CF_3COCHCO(C_6H_5)$)$_3$] *fac* complex 4 17
7) [Ru($CF_3COCHCO(C_6H_5)$)$_3$] *mer* complex 4 18
8) [Ru($CH_3COCHCOCF_3$)$_3$] *fac* complex 5 19
9) [Ru($CH_3COCHCOCF_3$)$_3$] *mer* complex 5 20
10) [Ru($CF_3COCHCOC(CH_3)_3$)$_3$] *fac* complex 6 21
11) [Ru($CF_3COCHCOC(CH_3)_3$)$_3$] *mer* complex 6 23
12) [Ru($(C_6H_5)COCHCO(C_6H_5)$)$_3$] complex 7 24
13) [Ru($CH_3COCHCO(C_6H_5)$)$_3$] *fac* complex 8 26
14) [Ru($CH_3COCHCO(C_6H_5)$)$_3$] *mer* complex 8 27
15) [Ru($CH_3COCHCOCH_3$)$_3$] complex 9 28
16) [Ru($C(CH_3)_3COCHCOC(CH_3)_3$)$_3$] complex 10 29
17) [Ru(EtCOCHCOEt)$_3$] complex 11 31
18) [Ru(PrCOCHCOPr)$_3$] complex 12 33
19) [Ru(BuCOCHCOBu)$_3$] complex 13 34
20) [Ru(*i*PrCOCHCO*i*Pr)$_3$] complex 14 36

Electronic energy data

DFT calculated electronic energies in eV of the [Ru(β-diketonato)$_3$] compounds 1 – 14, computed using the indicated methods.

No	PW91/STO -TZ2P	OLYP/STO -TZ2P	OPBE0/STO -TZ2P	B3LYP/STO -TZ2P	B3LYP/GTO-6-311G(d,p)/Lanl2dz
**1**	-270.14	-257.74	-351.20	-324.25	-79366.32
**2-fac**	-365.77	-349.59	-460.30	-426.03	-70530.79
**2-mer**	-365.75	-349.49	-460.20	-426.04	-70530.82
**3-fac**	-358.19	-341.93	-450.12	-415.73	-96897.22
**3-mer**	-358.19	-341.93	-450.12	-415.73	-96897.22
**4-fac**	-423.77	-404.91	-525.12	-487.68	-70711.87
**4-mer**	-423.78	-404.92	-525.15	-487.71	-70711.92
**5-fac**	-267.21	-255.23	-337.39	-313.08	-55055.98
**5-mer**	-267.16	-255.23	-337.35	-313.06	-55056.00
**6-fac**	-416.11	-397.45	-514.45	-478.79	-64686.43
**6-mer**	-416.11	-397.47	-514.47	-478.80	-64686.47
**7**	-263.82	-252.38	-698.67	-650.60	-62056.88
**8-fac**	-420.39	-402.06	-510.92	-476.03	-46401.06
**8-mer**	-420.40	-402.05	-510.89	-476.02	-46401.06
**9**	-263.84	-252.38	-323.15	-301.41	-30745.21
**10**	-561.39	-536.49	-676.96	-632.56	-50005.83
**11**	-363.02	-347.40	-441.28	-411.92	-37165.59
**12**	-462.48	-442.71	-559.73	-522.70	-43586.02
**13**	-561.84	-537.89	-678.08	-633.39	-50006.36
**14**	-462.41	-442.37	-559.45	-522.51	-43586.07

Example input files

(i) B3LYP/GTO-6-311G(d,p)/Lanl2dz (Gaussian 16)

```
%chk=Ru_acac_b3lyp_s12.chk
%mem=800mb
#p b3lyp/Gen pseudo=read
opt scf=(conver=8)
scrf=(solvent=acetonitrile,iefpcm)

Comment:

0 2
44       0.166123000       0.131242000      -0.074029000
8       -1.334704000      -0.596028000       1.112816000
8        0.130931000      -1.526701000      -1.273986000
8        0.212479000       1.823579000       1.115243000
8        1.550727000      -0.770815000       1.122479000
```

```
8         1.693523000      0.882720000     -1.250014000
8        -1.222012000      1.027858000     -1.270509000
6        -1.897408000     -1.725758000      0.936735000
6        -0.665840000     -2.507575000     -1.109733000
6        -0.659683000      2.740600000      1.103620000
6         2.778463000     -0.960442000      0.811787000
6         2.889207000      0.468178000     -1.235446000
6        -1.904408000      2.064493000     -0.954976000
6        -2.958080000     -2.056846000      1.961694000
6        -1.621574000     -2.644436000     -0.089461000
6        -0.523407000     -3.602071000     -2.142678000
6        -0.507355000      3.775409000      2.195076000
6        -1.703552000      2.875795000      0.163879000
6         3.536385000     -1.818697000      1.796551000
6         3.439604000     -0.433127000     -0.299616000
6         3.773497000      1.042619000     -2.318668000
6        -2.994529000      2.406957000     -1.942472000
1        -2.504087000     -2.061664000      2.956154000
1        -3.719669000     -1.272770000      1.958423000
1        -3.431350000     -3.021059000      1.777178000
1        -2.207056000     -3.553434000     -0.094540000
1         0.505520000     -3.970957000     -2.137421000
1        -1.206209000     -4.433333000     -1.967970000
1        -0.710945000     -3.182936000     -3.134770000
1         3.033204000     -2.784336000      1.893668000
1         4.572454000     -1.978001000      1.498255000
1         3.512524000     -1.342159000      2.780194000
1         4.486531000     -0.678404000     -0.411694000
1         3.377362000      0.748338000     -3.294339000
1         3.737568000      2.133742000     -2.270731000
1         4.807710000      0.709358000     -2.234834000
1         0.498700000      4.199724000      2.149835000
1        -1.242602000      4.576115000      2.117821000
1        -0.608244000      3.284833000      3.166940000
1        -2.360757000      3.726386000      0.279458000
1        -3.664662000      1.549943000     -2.049831000
1        -3.570000000      3.281690000     -1.640028000
1        -2.545962000      2.590419000     -2.922444000

C H O 0
6-311G(d,p)
****
Ru 0
Lanl2dz
****

Ru 0
lanl2dz
```

(ii) PW91/STO-TZ2P (ADF 2018)

```
TITLE Ru(beta-dik)3 project

COMMENT
  Input geom for Ru(acac)3
END

UNITS
  length Angstrom
END

Atoms Cartesian
Ru        0.000000000      0.000000000      0.000000000
O        -1.425730000     -0.781504000      1.206095000
O         0.036062000     -1.625470000     -1.206095000
O         0.036062000      1.625470000      1.206095000
O         1.389667000     -0.843966000      1.206095000
O         1.389667000      0.843966000     -1.206095000
O        -1.425730000      0.781504000     -1.206095000
C        -1.950719000     -1.945096000      1.032407000
C        -0.709143000     -2.661920000     -1.032407000
C        -0.709143000      2.661920000      1.032407000
C         2.659862000     -0.716824000      1.032407000
C         2.659862000      0.716824000     -1.032407000
C        -1.950719000      1.945096000     -1.032407000
C        -2.991841000     -2.336471000      2.056803000
C        -1.642499000     -2.844892000      0.000000000
C        -0.527523000     -3.759246000     -2.056803000
C        -0.303277952      3.644562693      2.107667080
C        -1.642499000      2.844892000      0.000000000
C         3.519364000     -1.422774000      2.056803000
C         3.284998000      0.000000000      0.000000000
C         3.519364000      1.422774000     -2.056803000
C        -2.991841000      2.336471000     -2.056803000
H        -3.118207000     -1.527972000      2.782453000
H        -3.954249000     -2.540755000      1.569529000
H        -2.687928000     -3.250519000      2.583717000
H        -2.187490000     -3.788843000      0.000000000
H         0.235841000     -3.464432000     -2.782453000
H        -0.223234000     -4.694858000     -1.569529000
H        -1.471068000     -3.953074000     -2.583717000
H         2.882366000     -1.936460000      2.782453000
H         4.177483000     -2.154103000      1.569529000
H         4.158996000     -0.702555000      2.583717000
H         4.310841378      0.368383122      0.000000027
H         2.882366000      1.936460000     -2.782453000
H         4.177483000      2.154103000     -1.569529000
H         4.158996000      0.702555000     -2.583717000
H         0.482513039      3.207217124      2.730137309
H         0.065339224      4.576341558      1.658994499
```

```
H       -1.164130576      3.900243062      2.739367273
H       -2.187490000      3.788843000      0.000000000
H       -3.118207000      1.527972000     -2.782453000
H       -3.954249000      2.540755000     -1.569529000
H       -2.687928000      3.250519000     -2.583717000
END

SYMMETRY nosym

Charge 0 1

unrestricted

GEOMETRY
  Iterations 1000
END

SCF
  Iterations 1000
END

RELATIVISTIC Scalar ZORA

BASIS
  Type TZ2P
  Core None
END

SOLVATION
  Radii
   Ru=1.950
   C=1.700
   H=1.350
   O=1.517
   F=1.425
   S=1.792
  Subend
  Solv name=Acetonitrile
END

XC
  gga PW91
END

endinput
```

(iii) OLYP/STO-TZ2P (ADF 2018)

```
TITLE Ru(beta-dik)3 project

COMMENT
  Input geom for Ru(acac)3
END

UNITS
  length Angstrom
END

Atoms Cartesian
Ru        0.000000000      0.000000000      0.000000000
O        -1.425730000     -0.781504000      1.206095000
O         0.036062000     -1.625470000     -1.206095000
O         0.036062000      1.625470000      1.206095000
O         1.389667000     -0.843966000      1.206095000
O         1.389667000      0.843966000     -1.206095000
O        -1.425730000      0.781504000     -1.206095000
C        -1.950719000     -1.945096000      1.032407000
C        -0.709143000     -2.661920000     -1.032407000
C        -0.709143000      2.661920000      1.032407000
C         2.659862000     -0.716824000      1.032407000
C         2.659862000      0.716824000     -1.032407000
C        -1.950719000      1.945096000     -1.032407000
C        -2.991841000     -2.336471000      2.056803000
C        -1.642499000     -2.844892000      0.000000000
C        -0.527523000     -3.759246000     -2.056803000
C        -0.303277952      3.644562693      2.107667080
C        -1.642499000      2.844892000      0.000000000
C         3.519364000     -1.422774000      2.056803000
C         3.284998000      0.000000000      0.000000000
C         3.519364000      1.422774000     -2.056803000
C        -2.991841000      2.336471000     -2.056803000
H        -3.118207000     -1.527972000      2.782453000
H        -3.954249000     -2.540755000      1.569529000
H        -2.687928000     -3.250519000      2.583717000
H        -2.187490000     -3.788843000      0.000000000
H         0.235841000     -3.464432000     -2.782453000
H        -0.223234000     -4.694858000     -1.569529000
H        -1.471068000     -3.953074000     -2.583717000
H         2.882366000     -1.936460000      2.782453000
H         4.177483000     -2.154103000      1.569529000
H         4.158996000     -0.702555000      2.583717000
H         4.310841378      0.368383122      0.000000027
H         2.882366000      1.936460000     -2.782453000
H         4.177483000      2.154103000     -1.569529000
H         4.158996000      0.702555000     -2.583717000
H         0.482513039      3.207217124      2.730137309
H         0.065339224      4.576341558      1.658994499
H        -1.164130576      3.900243062      2.739367273
```

```
H       -2.187490000      3.788843000      0.000000000
H       -3.118207000      1.527972000     -2.782453000
H       -3.954249000      2.540755000     -1.569529000
H       -2.687928000      3.250519000     -2.583717000
END

SYMMETRY nosym

Charge 0 1

unrestricted

GEOMETRY
  Iterations 1000
END

SCF
  Iterations 1000
END

RELATIVISTIC Scalar ZORA

BASIS
  Type TZ2P
  Core None
END

SOLVATION
  Radii
   Ru=1.950
   C=1.700
   H=1.350
   O=1.517
   F=1.425
   S=1.792
  Subend
  Solv name=Acetonitrile
END

XC
  gga OLYP
END

endinput
```

(iv) B3LYP/STO-TZ2P (ADF 2018)

```
TITLE Ru(beta-dik)3 project
```

```
COMMENT
  Input geom for Ru(acac)3
END

UNITS
  length Angstrom
END

Atoms Cartesian
Ru        0.000000000      0.000000000      0.000000000
O        -1.425730000     -0.781504000      1.206095000
O         0.036062000     -1.625470000     -1.206095000
O         0.036062000      1.625470000      1.206095000
O         1.389667000     -0.843966000      1.206095000
O         1.389667000      0.843966000     -1.206095000
O        -1.425730000      0.781504000     -1.206095000
C        -1.950719000     -1.945096000      1.032407000
C        -0.709143000     -2.661920000     -1.032407000
C        -0.709143000      2.661920000      1.032407000
C         2.659862000     -0.716824000      1.032407000
C         2.659862000      0.716824000     -1.032407000
C        -1.950719000      1.945096000     -1.032407000
C        -2.991841000     -2.336471000      2.056803000
C        -1.642499000     -2.844892000      0.000000000
C        -0.527523000     -3.759246000     -2.056803000
C        -0.303277952      3.644562693      2.107667080
C        -1.642499000      2.844892000      0.000000000
C         3.519364000     -1.422774000      2.056803000
C         3.284998000      0.000000000      0.000000000
C         3.519364000      1.422774000     -2.056803000
C        -2.991841000      2.336471000     -2.056803000
H        -3.118207000     -1.527972000      2.782453000
H        -3.954249000     -2.540755000      1.569529000
H        -2.687928000     -3.250519000      2.583717000
H        -2.187490000     -3.788843000      0.000000000
H         0.235841000     -3.464432000     -2.782453000
H        -0.223234000     -4.694858000     -1.569529000
H        -1.471068000     -3.953074000     -2.583717000
H         2.882366000     -1.936460000      2.782453000
H         4.177483000     -2.154103000      1.569529000
H         4.158996000     -0.702555000      2.583717000
H         4.310841378      0.368383122      0.000000027
H         2.882366000      1.936460000     -2.782453000
H         4.177483000      2.154103000     -1.569529000
H         4.158996000      0.702555000     -2.583717000
H         0.482513039      3.207217124      2.730137309
H         0.065339224      4.576341558      1.658994499
H        -1.164130576      3.900243062      2.739367273
H        -2.187490000      3.788843000      0.000000000
H        -3.118207000      1.527972000     -2.782453000
H        -3.954249000      2.540755000     -1.569529000
```

```
H       -2.687928000      3.250519000     -2.583717000
END

SYMMETRY nosym

Charge 0 1

unrestricted

GEOMETRY
  Iterations 1000
END

SCF
  Iterations 1000
END

RELATIVISTIC Scalar ZORA

BASIS
  Type TZ2P
  Core None
END

SOLVATION
  Radii
   Ru=1.950
   C=1.700
   H=1.350
   O=1.517
   F=1.425
   S=1.792
  Subend
  Solv name=Acetonitrile
END

XC
  Hybrid B3LYP
END

endinput
```

(v) OPBE0/STO-TZ2P (ADF 2018)

```
TITLE Ru(beta-dik)3 project

COMMENT
  Input geom for Ru(acac)3
END
```

```
UNITS
  length Angstrom
END

Atoms Cartesian
Ru        0.000000000      0.000000000      0.000000000
O        -1.425730000     -0.781504000      1.206095000
O         0.036062000     -1.625470000     -1.206095000
O         0.036062000      1.625470000      1.206095000
O         1.389667000     -0.843966000      1.206095000
O         1.389667000      0.843966000     -1.206095000
O        -1.425730000      0.781504000     -1.206095000
C        -1.950719000     -1.945096000      1.032407000
C        -0.709143000     -2.661920000     -1.032407000
C        -0.709143000      2.661920000      1.032407000
C         2.659862000     -0.716824000      1.032407000
C         2.659862000      0.716824000     -1.032407000
C        -1.950719000      1.945096000     -1.032407000
C        -2.991841000     -2.336471000      2.056803000
C        -1.642499000     -2.844892000      0.000000000
C        -0.527523000     -3.759246000     -2.056803000
C        -0.303277952      3.644562693      2.107667080
C        -1.642499000      2.844892000      0.000000000
C         3.519364000     -1.422774000      2.056803000
C         3.284998000      0.000000000      0.000000000
C         3.519364000      1.422774000     -2.056803000
C        -2.991841000      2.336471000     -2.056803000
H        -3.118207000     -1.527972000      2.782453000
H        -3.954249000     -2.540755000      1.569529000
H        -2.687928000     -3.250519000      2.583717000
H        -2.187490000     -3.788843000      0.000000000
H         0.235841000     -3.464432000     -2.782453000
H        -0.223234000     -4.694858000     -1.569529000
H        -1.471068000     -3.953074000     -2.583717000
H         2.882366000     -1.936460000      2.782453000
H         4.177483000     -2.154103000      1.569529000
H         4.158996000     -0.702555000      2.583717000
H         4.310841378      0.368383122      0.000000027
H         2.882366000      1.936460000     -2.782453000
H         4.177483000      2.154103000     -1.569529000
H         4.158996000      0.702555000     -2.583717000
H         0.482513039      3.207217124      2.730137309
H         0.065339224      4.576341558      1.658994499
H        -1.164130576      3.900243062      2.739367273
H        -2.187490000      3.788843000      0.000000000
H        -3.118207000      1.527972000     -2.782453000
H        -3.954249000      2.540755000     -1.569529000
H        -2.687928000      3.250519000     -2.583717000
END
```

```
SYMMETRY nosym

Charge 0 1

unrestricted

GEOMETRY
  Iterations 1000
END

SCF
  Iterations 1000
END

RELATIVISTIC Scalar ZORA

BASIS
  Type TZ2P
  Core None
END

SOLVATION
  Radii
   Ru=1.950
   C=1.700
   H=1.350
   O=1.517
   F=1.425
   S=1.792
  Subend
  Solv name=Acetonitrile
END

XC
  Hybrid OPBE0
END

endinput
```

Optimized Cartesian coordinates (Å) for PW91/TZ2P

1) [Ru($CF_3COCHCOCF_3$)$_3$] complex 1

Ru	-0.086850000	0.001785000	-0.001993000
O	-1.528570000	0.835164000	-1.150220000
O	-0.066947000	1.583815000	1.232649000
O	-0.068713000	-1.581407000	-1.238210000
O	1.282155000	0.865776000	-1.173358000
O	1.274621000	-0.871296000	1.173057000

O	-1.534483000	-0.829540000	1.145257000
C	-1.906012000	2.045033000	-1.047624000
C	-0.667268000	2.692708000	0.975283000
C	-0.670880000	-2.688938000	-0.980445000
C	2.544729000	0.729279000	-0.987163000
C	2.538227000	-0.737543000	0.994745000
C	-1.907840000	-2.040666000	1.044110000
C	-1.516872000	2.987365000	-0.082753000
C	-1.519090000	-2.983081000	0.078906000
C	3.195823000	-0.003554000	0.006194000
H	-1.951035000	3.979189000	-0.131829000
H	4.280070000	-0.005291000	0.008281000
H	-1.950690000	-3.975885000	0.130295000
C	-0.373251000	3.771049000	2.040488000
F	-1.038937000	4.924786000	1.811110000
F	-0.719783000	3.324477000	3.274205000
F	0.956726000	4.052520000	2.063535000
C	-2.968965000	2.425102000	-2.105302000
F	-2.535699000	2.104361000	-3.349206000
F	-4.116308000	1.731770000	-1.876758000
F	-3.267917000	3.743008000	-2.091374000
C	3.357941000	-1.512196000	2.046391000
F	3.054729000	-1.073925000	3.295955000
F	3.058707000	-2.836423000	1.992839000
F	4.692142000	-1.378354000	1.869221000
C	-0.383705000	-3.765246000	-2.049964000
F	0.948274000	-4.031328000	-2.098572000
F	-1.032633000	-4.926272000	-1.808020000
F	-0.759300000	-3.322741000	-3.277023000
C	3.371931000	1.500694000	-2.035335000
F	4.704759000	1.345157000	-1.865263000
F	3.056768000	1.079479000	-3.287363000
F	3.093450000	2.829381000	-1.967546000
C	-2.963045000	-2.425058000	2.108499000
F	-4.110290000	-1.726863000	1.895800000
F	-3.265220000	-3.741989000	2.088587000
F	-2.516785000	-2.113590000	3.350527000

2) **[Ru(CF_3COCHCO(C_4H_3O))$_3$] *fac* complex 2**

Ru	-0.132556000	-0.208690000	-0.091993000
O	1.005716000	-1.260006000	1.168166000
C	1.510975000	-2.427648000	0.896555000
C	1.157311000	-3.236941000	-0.204681000
H	1.634213000	-4.208077000	-0.277781000
C	0.203968000	-2.907625000	-1.157880000
O	-0.474408000	-1.824721000	-1.275849000
C	2.487874000	-2.894422000	1.854578000
C	2.994313000	-2.324343000	3.002776000
H	2.711162000	-1.361066000	3.407778000
C	3.938621000	-3.240240000	3.529844000

H	4.535615000	-3.127832000	4.426964000
C	3.953965000	-4.311055000	2.677119000
H	4.508092000	-5.241132000	2.663024000
O	3.079656000	-4.125636000	1.650867000
O	0.110644000	1.364481000	1.121083000
C	1.101436000	2.192226000	1.032801000
C	2.147588000	2.125158000	0.075944000
H	2.914806000	2.889687000	0.135420000
C	2.263467000	1.180457000	-0.926264000
O	1.485014000	0.188669000	-1.209816000
C	1.093937000	3.257293000	2.013221000
C	0.224464000	3.557372000	3.038184000
H	-0.667125000	2.999084000	3.294342000
C	0.727112000	4.721792000	3.671627000
H	0.301643000	5.246120000	4.519335000
C	1.869499000	5.063710000	2.999717000
H	2.587400000	5.865358000	3.114295000
O	-1.224487000	0.928458000	-1.338929000
O	2.114444000	4.187840000	1.986247000
O	-1.753668000	-0.662900000	1.030666000
C	-2.837246000	0.029930000	1.038835000
C	-3.170046000	1.036083000	0.083392000
H	-4.132948000	1.522600000	0.192785000
C	-2.393368000	1.375191000	-1.000911000
C	-3.772140000	-0.289046000	2.091422000
C	-3.689052000	-1.149278000	3.165196000
H	-2.841931000	-1.779126000	3.405367000
C	-4.908923000	-1.033086000	3.875596000
H	-5.198545000	-1.558285000	4.777535000
C	-5.662755000	-0.111147000	3.198464000
H	-6.650183000	0.301681000	3.358741000
O	-4.994170000	0.355767000	2.110127000
C	-2.918732000	2.410268000	-2.010464000
F	-2.078282000	3.482129000	-2.077915000
F	-2.984976000	1.876033000	-3.262387000
F	-4.152404000	2.880376000	-1.702072000
C	3.469049000	1.243623000	-1.875403000
F	4.248089000	2.336350000	-1.680964000
F	4.262591000	0.144495000	-1.713198000
F	3.065679000	1.255095000	-3.175828000
C	-0.167043000	-3.943464000	-2.235132000
F	0.035117000	-3.435468000	-3.483694000
F	0.547328000	-5.092100000	-2.142117000
F	-1.486700000	-4.275374000	-2.148424000

3) [Ru($CF_3COCHCO(C_4H_3O))_3$] *mer* complex 2

Ru	2.455848000	2.088267000	10.632732000
O	0.792819000	1.646555000	11.649117000
C	0.705242000	1.687012000	12.947507000
C	1.796674000	1.738514000	13.841342000

H	1.571131000	1.695881000	14.900952000
C	3.129602000	1.787565000	13.461356000
O	3.623939000	1.880750000	12.281118000
C	-0.644877000	1.648683000	13.459842000
C	-1.867700000	1.644394000	12.825179000
H	-2.023110000	1.643947000	11.753883000
C	-2.855248000	1.650485000	13.843560000
H	-3.931030000	1.653716000	13.718841000
C	-2.179414000	1.654251000	15.034108000
H	-2.498011000	1.659439000	16.068446000
C	4.223811000	1.683965000	14.541412000
F	3.731083000	1.674487000	15.804551000
F	5.099378000	2.723006000	14.451997000
F	4.945753000	0.537803000	14.376037000
O	-0.834067000	1.653709000	14.827885000
O	2.213401000	4.020641000	11.090860000
C	1.474260000	4.828296000	10.393621000
C	0.749253000	4.492254000	9.222934000
H	0.171171000	5.285837000	8.763373000
C	0.726923000	3.249764000	8.615801000
O	1.332039000	2.167987000	8.968012000
C	1.424184000	6.185465000	10.890797000
C	1.994492000	6.785696000	11.992299000
H	2.621693000	6.294318000	12.725215000
C	1.596374000	8.146399000	11.966182000
H	1.855699000	8.922372000	12.676928000
C	0.810337000	8.297297000	10.855561000
H	0.286798000	9.140591000	10.423398000
C	-0.102741000	3.052523000	7.333463000
F	-0.966676000	2.007886000	7.471325000
F	-0.835479000	4.143553000	6.998342000
F	0.705278000	2.762979000	6.273135000
O	4.086857000	2.620595000	9.586578000
O	0.689217000	7.117873000	10.185736000
O	2.707971000	0.134802000	10.176906000
C	3.379286000	-0.270271000	9.156338000
C	4.321903000	0.518631000	8.432069000
H	4.874184000	0.025723000	7.639941000
C	4.625572000	1.831500000	8.712174000
C	3.153565000	-1.641139000	8.769837000
C	2.256360000	-2.587271000	9.220125000
H	1.537584000	-2.446826000	10.017921000
C	2.456498000	-3.745350000	8.428921000
H	1.926435000	-4.688119000	8.494803000
C	3.458403000	-3.440390000	7.545714000
H	3.945924000	-3.996455000	6.755922000
O	3.898666000	-2.167641000	7.733051000
C	5.754401000	2.532946000	7.940411000
F	5.278553000	3.627517000	7.280200000
F	6.719579000	2.972658000	8.797095000
F	6.355065000	1.734799000	7.024614000

4) **[Ru($CF_3COCHCO(C_4H_3S)$)$_3$] *fac* complex 3**

Ru	9.199957000	5.356699000	0.666822000
S	9.482376000	8.480373000	4.252643000
F	6.335319000	7.723160000	-2.332530000
F	5.140072000	6.183110000	-1.331208000
O	9.164383000	6.996682000	1.831644000
F	4.975802000	8.275950000	-0.703655000
O	7.780110000	6.126795000	-0.535304000
C	8.164338000	7.797425000	1.943814000
C	7.088063000	7.853815000	1.006604000
H	6.315758000	8.597788000	1.169064000
C	6.998844000	7.079569000	-0.129383000
C	5.850133000	7.326982000	-1.121079000
C	8.200804000	8.682349000	3.088313000
C	7.718679000	10.306181000	4.688198000
H	7.176629000	11.121280000	5.160168000
C	8.860049000	9.743933000	5.222782000
H	9.367428000	10.009414000	6.144090000
C	7.340696000	9.703115000	3.472190000
H	6.470582000	10.004670000	2.896432000
S	6.214723000	4.191207000	4.237762000
F	8.508191000	1.915069000	-2.430432000
F	10.185198000	1.183958000	-1.223988000
O	7.824453000	4.602550000	1.904069000
F	8.171433000	0.383056000	-0.899521000
O	9.284078000	3.711299000	-0.471731000
C	7.431994000	3.371380000	1.908014000
C	7.781410000	2.415275000	0.912957000
H	7.349379000	1.424352000	0.999124000
C	8.620392000	2.643375000	-0.159958000
C	8.863464000	1.516295000	-1.176002000
C	6.584441000	2.995946000	3.023315000
C	5.269204000	1.816035000	4.544117000
H	4.741258000	0.963924000	4.964568000
C	5.297951000	3.059179000	5.138404000
H	4.825725000	3.369095000	6.064546000
C	6.001868000	1.776965000	3.339783000
H	6.102064000	0.885085000	2.727793000
S	11.639571000	3.613652000	4.368288000
F	12.486684000	6.327010000	-2.540429000
F	12.522940000	8.122539000	-1.284582000
O	10.537057000	4.633398000	1.956108000
F	14.097369000	6.610483000	-1.080781000
O	10.639456000	6.202483000	-0.497021000
C	11.825224000	4.780284000	1.886954000
C	12.504732000	5.431968000	0.830976000
H	13.586564000	5.480566000	0.885631000
C	11.890857000	6.067757000	-0.240329000
C	12.766697000	6.779965000	-1.286356000

C	12.550577000	4.251585000	3.025936000
C	14.210755000	3.602439000	4.527494000
H	15.218023000	3.453028000	4.906637000
C	13.075964000	3.253132000	5.229655000
H	13.011059000	2.802674000	6.214628000
C	13.914014000	4.173285000	3.273144000
H	14.673074000	4.512776000	2.572707000

5) $[Ru(CF_3COCHCO(C_4H_3S))_3]$ *mer* complex 3

Ru	9.199957000	5.356699000	0.666822000
S	9.482376000	8.480373000	4.252643000
F	6.335319000	7.723160000	-2.332530000
F	5.140072000	6.183110000	-1.331208000
O	9.164383000	6.996682000	1.831644000
F	4.975802000	8.275950000	-0.703655000
O	7.780110000	6.126795000	-0.535304000
C	8.164338000	7.797425000	1.943814000
C	7.088063000	7.853815000	1.006604000
H	6.315758000	8.597788000	1.169064000
C	6.998844000	7.079569000	-0.129383000
C	5.850133000	7.326982000	-1.121079000
C	8.200804000	8.682350000	3.088313000
C	7.718679000	10.306181000	4.688198000
H	7.176629000	11.121280000	5.160168000
C	8.860049000	9.743933000	5.222782000
H	9.367428000	10.009414000	6.144090000
C	7.340696000	9.703115000	3.472190000
H	6.470582000	10.004670000	2.896432000
S	6.214723000	4.191207000	4.237762000
F	8.508191000	1.915069000	-2.430432000
F	10.185198000	1.183958000	-1.223988000
O	7.824453000	4.602550000	1.904069000
F	8.171433000	0.383056000	-0.899521000
O	9.284078000	3.711299000	-0.471731000
C	7.431994000	3.371380000	1.908014000
C	7.781410000	2.415275000	0.912957000
H	7.349379000	1.424352000	0.999124000
C	8.620392000	2.643375000	-0.159958000
C	8.863464000	1.516295000	-1.176002000
C	6.584441000	2.995946000	3.023315000
C	5.269204000	1.816035000	4.544117000
H	4.741258000	0.963924000	4.964568000
C	5.297951000	3.059179000	5.138404000
H	4.825725000	3.369095000	6.064546000
C	6.001868000	1.776965000	3.339783000
H	6.102064000	0.885085000	2.727794000
S	11.639571000	3.613652000	4.368288000
F	12.486684000	6.327010000	-2.540429000
F	12.522940000	8.122539000	-1.284582000
O	10.537057000	4.633398000	1.956108000

F	14.097369000	6.610483000	-1.080781000
O	10.639456000	6.202483000	-0.497021000
C	11.825224000	4.780284000	1.886954000
C	12.504732000	5.431968000	0.830976000
H	13.586564000	5.480566000	0.885631000
C	11.890857000	6.067757000	-0.240329000
C	12.766697000	6.779965000	-1.286356000
C	12.550577000	4.251585000	3.025936000
C	14.210755000	3.602439000	4.527494000
H	15.218023000	3.453028000	4.906637000
C	13.075964000	3.253132000	5.229655000
H	13.011059000	2.802674000	6.214628000
C	13.914014000	4.173285000	3.273144000
H	14.673074000	4.512776000	2.572707000

6) $[Ru(CF_3COCHCO(C_6H_5))_3]$ *fac* complex 4

Ru	0.101986000	0.197649000	1.213630000
O	1.401810000	1.230836000	0.067097000
O	1.574440000	-0.378782000	2.470668000
O	-1.355401000	0.679064000	-0.051229000
O	0.464255000	-1.360888000	0.018238000
O	-1.223277000	-0.794352000	2.340489000
O	-0.212159000	1.847781000	2.340839000
C	2.675080000	1.085501000	0.067086000
C	2.820432000	-0.285473000	2.140700000
C	-2.272943000	1.570725000	0.154557000
C	-0.223255000	-2.450879000	-0.002774000
C	-1.645963000	-1.964869000	1.990800000
C	-1.248945000	2.585613000	2.184642000
C	3.387774000	0.335758000	1.045669000
C	-2.250868000	2.491935000	1.223544000
C	-1.247958000	-2.758865000	0.929885000
H	4.464733000	0.271865000	0.945580000
H	-1.772078000	-3.699523000	0.805674000
H	-3.034771000	3.240005000	1.261631000
C	-3.356684000	1.594471000	-0.857073000
C	-4.585296000	2.236767000	-0.622405000
C	-3.161701000	0.929467000	-2.082366000
C	-5.588560000	2.213318000	-1.588404000
C	-4.163405000	0.917449000	-3.048020000
C	-5.380736000	1.558101000	-2.804211000
H	-2.213688000	0.430833000	-2.268878000
H	-6.538764000	2.705126000	-1.388418000
H	-3.994662000	0.406446000	-3.994774000
H	-6.166996000	1.544242000	-3.558652000
C	3.393493000	1.770406000	-1.032147000
C	2.679494000	2.109696000	-2.196180000
C	4.756724000	2.098116000	-0.948533000
C	3.317258000	2.751640000	-3.252683000
C	5.387758000	2.751949000	-2.004385000

C	4.672377000	3.076324000	-3.158699000
H	1.625269000	1.851752000	-2.264166000
H	5.324871000	1.871937000	-0.049346000
H	2.757726000	2.997613000	-4.153472000
H	6.441196000	3.012844000	-1.923459000
H	5.170731000	3.582329000	-3.985108000
C	0.109518000	-3.388660000	-1.100859000
C	-0.270377000	-4.741799000	-1.078710000
C	0.830916000	-2.906072000	-2.208042000
C	0.057647000	-5.583312000	-2.137627000
C	1.149194000	-3.748158000	-3.266950000
C	0.764543000	-5.090721000	-3.235699000
H	-0.804733000	-5.153679000	-0.225684000
H	1.127133000	-1.860291000	-2.230065000
H	-0.236992000	-6.630431000	-2.103174000
H	1.697772000	-3.356823000	-4.122183000
H	1.015617000	-5.750607000	-4.064534000
H	-4.777721000	2.735871000	0.324507000
C	3.729355000	-0.990206000	3.163607000
C	-1.283206000	3.760283000	3.180566000
C	-2.751957000	-2.466512000	2.934467000
F	-2.302957000	-2.518910000	4.219794000
F	-3.817373000	-1.615582000	2.918410000
F	-3.212776000	-3.700502000	2.613198000
F	3.538866000	-0.477909000	4.411326000
F	3.434549000	-2.319941000	3.227667000
F	5.049176000	-0.880825000	2.874624000
F	-0.380499000	4.718476000	2.811093000
F	-0.946304000	3.354806000	4.431489000
F	-2.498635000	4.358049000	3.254876000

7) [Ru(CF_3COCHCO(C_6H_5))$_3$] *mer* complex 4

Ru	3.136400000	4.044603000	2.083706000
O	4.435073000	4.000915000	0.575297000
O	1.938265000	5.381357000	1.197642000
O	4.289529000	2.689147000	3.035286000
O	4.260862000	5.505764000	2.870133000
O	1.903912000	4.032383000	3.676747000
O	2.025597000	2.596776000	1.231812000
C	4.397579000	4.760861000	-0.467082000
C	2.225804000	5.881647000	0.042285000
C	4.326032000	1.428390000	2.807253000
C	3.899212000	6.166286000	3.922786000
C	1.881479000	4.890506000	4.629792000
C	2.408225000	1.361605000	1.220891000
C	3.334879000	5.655368000	-0.756348000
C	3.455533000	0.766837000	1.894058000
C	2.821422000	5.954252000	4.756521000
H	3.371681000	6.191011000	-1.698376000
H	2.738353000	6.601267000	5.621330000

H	3.595771000	-0.297630000	1.741720000
C	0.836808000	4.683618000	5.657490000
C	0.213751000	3.426146000	5.749385000
C	0.437872000	5.705853000	6.535955000
C	-0.772085000	3.194547000	6.700488000
C	-0.558985000	5.472298000	7.478623000
C	-1.163106000	4.217772000	7.568259000
H	0.522270000	2.632372000	5.072776000
H	0.883137000	6.696115000	6.472666000
H	-1.237734000	2.212557000	6.768115000
H	-0.867362000	6.275534000	8.145487000
H	-1.938169000	4.037134000	8.311892000
C	5.328145000	0.663059000	3.582382000
C	5.242713000	-0.730766000	3.746522000
C	6.388245000	1.356242000	4.196956000
C	6.191471000	-1.409415000	4.504894000
C	7.337310000	0.672782000	4.946764000
C	7.241745000	-0.712546000	5.105128000
H	6.459600000	2.433988000	4.069139000
H	6.107269000	-2.487356000	4.633274000
H	8.156842000	1.219546000	5.410277000
H	7.983419000	-1.246776000	5.696387000
C	5.558273000	4.630975000	-1.377469000
C	6.409407000	3.519355000	-1.240703000
C	5.862426000	5.600592000	-2.348215000
C	7.527354000	3.379756000	-2.054306000
C	6.987258000	5.459864000	-3.156048000
C	7.822015000	4.350864000	-3.014117000
H	6.179376000	2.765676000	-0.490590000
H	5.240094000	6.484977000	-2.461346000
H	8.173705000	2.511435000	-1.939466000
H	7.217399000	6.225470000	-3.895718000
H	8.701055000	4.244137000	-3.647311000
H	4.420547000	-1.290723000	3.307970000
C	1.115140000	6.826818000	-0.450484000
C	1.499546000	0.495521000	0.325564000
C	4.884001000	7.309016000	4.229251000
F	1.424977000	7.464195000	-1.606249000
F	-0.038722000	6.129786000	-0.665428000
F	0.836042000	7.779839000	0.481107000
F	0.210189000	0.538168000	0.764038000
F	1.872909000	-0.806566000	0.283442000
F	1.503035000	0.963313000	-0.954274000
F	4.889201000	8.219392000	3.213441000
F	6.155037000	6.833638000	4.345771000
F	4.592684000	7.978716000	5.371246000

8) $[Ru(CH_3COCHCOCF_3)_3]$ *fac* complex 5

Ru	-0.142002000	-0.003873000	-0.082683000
O	-1.568915000	-0.812079000	1.106114000

O	-0.168559000	-1.597659000	-1.319320000
O	-0.049681000	1.554190000	1.168876000
O	1.193707000	-0.905124000	1.101809000
O	1.240737000	0.844262000	-1.263986000
O	-1.581141000	0.878045000	-1.213920000
C	-1.915117000	-2.039987000	1.103636000
C	-0.717549000	-2.715839000	-0.972974000
C	-0.588320000	2.710613000	0.957929000
C	2.467469000	-0.755132000	1.004459000
C	2.499127000	0.723681000	-1.007755000
C	-1.895583000	2.109052000	-1.071628000
C	-2.860438000	-2.476708000	2.182207000
C	-1.491809000	-2.994655000	0.133598000
C	-0.471730000	-3.822075000	-2.014711000
C	-0.260337000	3.752670000	1.984579000
C	-1.449217000	3.015833000	-0.110840000
C	3.283623000	-1.499355000	2.019068000
C	3.117440000	0.020474000	0.015430000
C	3.358839000	1.493327000	-2.024181000
C	-2.952664000	2.559551000	-2.101142000
H	-3.127208000	-1.633002000	2.822725000
H	-3.767237000	-2.911069000	1.741660000
H	-2.390054000	-3.261963000	2.788448000
H	-1.875719000	-4.003968000	0.242229000
H	2.737244000	-1.565754000	2.965147000
H	3.459870000	-2.524080000	1.661762000
H	4.256124000	-1.025220000	2.178591000
H	4.201413000	0.053715000	0.058468000
H	0.829260000	3.861320000	2.062224000
H	-0.708243000	4.719090000	1.744128000
H	-0.621245000	3.420698000	2.967016000
H	-1.843050000	4.026035000	-0.152704000
F	-2.544453000	2.278965000	-3.368291000
F	-4.125032000	1.890947000	-1.899469000
F	-3.225202000	3.885615000	-2.042556000
F	3.109408000	1.060181000	-3.291952000
F	3.064961000	2.824262000	-1.992272000
F	4.691025000	1.366575000	-1.805028000
F	0.848611000	-3.912278000	-2.326544000
F	-1.140403000	-3.542707000	-3.172193000
F	-0.877950000	-5.046572000	-1.598806000

9) [Ru($CH_3COCHCOCF_3$)$_3$] *mer* complex 5

Ru	-0.180050000	0.019272000	-0.015851000
O	-1.608399000	-0.818159000	1.163303000
O	-0.164864000	-1.560107000	-1.251905000
O	-0.129550000	1.585341000	1.237759000
O	1.171300000	-0.884693000	1.152963000
O	1.185237000	0.885309000	-1.198224000
O	-1.605690000	0.887326000	-1.186496000

C	-1.887440000	-2.061052000	1.095634000
C	-0.661722000	-2.720234000	-0.969329000
C	-0.661046000	2.740141000	0.997220000
C	2.443482000	-0.807287000	0.980550000
C	2.448361000	0.697900000	-1.011056000
C	-1.894858000	2.123859000	-1.078229000
C	-2.860423000	-2.565360000	2.187291000
C	-1.457332000	-2.999065000	0.154616000
C	-0.357245000	-3.798167000	-1.964432000
C	-0.373351000	3.791034000	2.025438000
C	-1.474615000	3.036881000	-0.107891000
C	3.290500000	-1.557535000	1.965561000
C	3.079974000	-0.064591000	-0.043431000
C	3.292884000	1.458275000	-2.046738000
C	-2.865798000	2.656358000	-2.158342000
H	-1.819998000	-4.017202000	0.263059000
H	0.730752000	-3.895863000	-2.075232000
H	-0.781242000	-4.759516000	-1.666831000
H	-0.757592000	-3.513069000	-2.945399000
H	2.666779000	-2.201732000	2.593097000
H	4.043279000	-2.160575000	1.445005000
H	3.830095000	-0.847724000	2.607039000
H	4.165302000	-0.095664000	-0.060817000
H	0.712966000	3.921877000	2.121336000
H	-0.835446000	4.747609000	1.772711000
H	-0.744783000	3.454868000	3.002235000
H	-1.855384000	4.051696000	-0.180894000
F	-3.245368000	1.692885000	-3.023635000
F	-3.993432000	3.179341000	-1.597170000
F	-2.280043000	3.656338000	-2.879845000
F	2.981572000	1.055298000	-3.311145000
F	3.045055000	2.796767000	-1.981802000
F	4.628000000	1.283913000	-1.881662000
F	-3.225321000	-1.584333000	3.038972000
F	-3.996510000	-3.082217000	1.637187000
F	-2.285524000	-3.562158000	2.921097000

10) $[Ru(CF_3COCHCOC(CH_3)_3)_3]$ *fac* complex 6

Ru	-0.041854000	0.140605000	-0.133336000
O	1.397978000	-0.640533000	-1.289915000
O	-0.158335000	-1.464033000	1.054342000
O	-0.006097000	1.829487000	-1.263877000
O	-1.430268000	-0.657049000	-1.364568000
O	-1.467648000	0.981042000	1.022339000
O	1.327873000	0.853974000	1.131369000
C	1.928889000	-1.784566000	-1.010253000
C	0.608328000	-2.487839000	0.992936000
C	0.843927000	2.765172000	-1.056881000
C	-2.669757000	-0.752937000	-1.007298000
C	-2.700959000	0.669223000	1.038997000

C	2.004601000	1.938438000	0.987829000
C	1.621734000	-2.663553000	0.015088000
C	1.803843000	2.866536000	-0.054749000
C	-3.305141000	-0.204221000	0.088407000
H	2.203438000	-3.578347000	0.062614000
H	-4.367686000	-0.397862000	0.190993000
H	2.419372000	3.759595000	-0.053658000
C	3.072479000	2.206083000	2.056220000
C	2.766575000	3.546078000	2.760324000
C	4.452298000	2.281359000	1.367018000
H	4.687366000	1.342493000	0.850193000
H	4.505214000	3.099104000	0.639493000
H	5.223594000	2.453655000	2.128689000
H	2.789761000	4.392449000	2.065320000
H	3.523393000	3.723920000	3.535163000
H	1.781880000	3.522474000	3.244168000
C	3.091030000	1.079597000	3.101493000
H	3.318151000	0.110117000	2.644546000
H	3.865373000	1.299198000	3.847016000
H	2.128787000	0.993266000	3.618746000
C	0.384162000	-3.569660000	2.057767000
C	-0.747559000	-3.163430000	3.011814000
C	1.687961000	-3.754661000	2.868546000
H	2.522118000	-4.085899000	2.240404000
H	1.978133000	-2.821891000	3.367365000
H	1.522832000	-4.517249000	3.640463000
H	-0.517914000	-2.227324000	3.533202000
H	-0.881838000	-3.952154000	3.762068000
H	-1.696024000	-3.029818000	2.478904000
C	0.011302000	-4.896744000	1.362290000
H	-0.910865000	-4.790509000	0.776929000
H	0.804797000	-5.251790000	0.695234000
H	-0.157062000	-5.666600000	2.126432000
C	-3.558490000	1.305703000	2.136874000
C	-4.707675000	2.111138000	1.493144000
C	-2.710251000	2.241731000	3.011774000
H	-1.884325000	1.704676000	3.492173000
H	-2.284005000	3.063189000	2.425574000
H	-3.346194000	2.669820000	3.795326000
H	-4.318383000	2.910169000	0.850971000
H	-5.308221000	2.572035000	2.286903000
H	-5.372359000	1.477795000	0.892682000
C	-4.141264000	0.175324000	3.013840000
H	-3.343646000	-0.420001000	3.474368000
H	-4.741321000	0.621882000	3.816300000
H	-4.790467000	-0.498483000	2.442069000
C	-3.482144000	-1.603201000	-2.001458000
F	-3.389209000	-1.099890000	-3.263083000
F	-2.997216000	-2.877790000	-2.041544000
F	-4.799220000	-1.681162000	-1.689343000
C	0.709854000	3.903845000	-2.087639000

F	1.493525000	4.974996000	-1.810778000
F	1.050646000	3.462023000	-3.333290000
F	-0.575735000	4.344376000	-2.157036000
C	3.047857000	-2.140474000	-2.004458000
F	4.032763000	-1.196941000	-1.974715000
F	3.628508000	-3.340314000	-1.754602000
F	2.568794000	-2.175644000	-3.279579000

11) [Ru($CF_3COCHCOC(CH_3)_3)_3$] *mer* complex 6

Ru	0.071376000	0.012262000	-0.046897000
O	1.541191000	-0.848543000	-1.132334000
O	0.003720000	-1.530840000	1.234589000
O	0.074020000	1.572810000	-1.324624000
O	-1.271146000	-0.876605000	-1.236205000
O	-1.311242000	0.892146000	1.103026000
O	1.486394000	0.875231000	1.106131000
C	1.935883000	-2.054767000	-1.035502000
C	0.604256000	-2.650034000	0.986814000
C	0.673126000	2.684311000	-1.048567000
C	-2.538550000	-0.752549000	-1.023268000
C	-2.575961000	0.716107000	0.999797000
C	1.897551000	2.077730000	1.034093000
C	1.468697000	-2.964424000	-0.040330000
C	1.493018000	2.991547000	0.017601000
C	-3.191387000	-0.056017000	-0.019798000
H	1.879291000	-3.968684000	-0.048386000
H	-4.275218000	-0.105009000	-0.020039000
H	1.913572000	3.991084000	0.045647000
C	2.995257000	-2.518736000	-2.044320000
C	4.233165000	-3.048031000	-1.289611000
C	3.413885000	-1.357291000	-2.954783000
H	2.561468000	-0.955786000	-3.513461000
H	3.857536000	-0.536702000	-2.379847000
H	4.160562000	-1.717785000	-3.673814000
H	4.670074000	-2.270359000	-0.651580000
H	4.992618000	-3.355775000	-2.020895000
H	3.999382000	-3.916549000	-0.664333000
C	2.383189000	-3.649363000	-2.900777000
H	1.498273000	-3.297618000	-3.445012000
H	3.127586000	-3.982992000	-3.636570000
H	2.095433000	-4.515376000	-2.294363000
C	2.910504000	2.526095000	2.097111000
C	2.295152000	3.695502000	2.898708000
C	4.207463000	2.995956000	1.404808000
H	4.653827000	2.188737000	0.811523000
H	4.039222000	3.855880000	0.747349000
H	4.933436000	3.296079000	2.172249000
H	2.079158000	4.562194000	2.264221000
H	3.008965000	4.011153000	3.671281000
H	1.366759000	3.389426000	3.396149000

C	3.235899000	1.369982000	3.051358000
H	3.682926000	0.522719000	2.518389000
H	3.952567000	1.719658000	3.805614000
H	2.339478000	1.009121000	3.567952000
C	-3.446199000	1.390383000	2.067937000
C	-4.430486000	2.367072000	1.389721000
C	-2.574874000	2.163776000	3.067340000
H	-1.867103000	1.502236000	3.579029000
H	-2.000918000	2.955849000	2.573608000
H	-3.222844000	2.625695000	3.821534000
H	-3.893765000	3.152023000	0.843036000
H	-5.045481000	2.848029000	2.160426000
H	-5.103757000	1.858115000	0.691023000
C	-4.232883000	0.296475000	2.823839000
H	-3.552538000	-0.418590000	3.302111000
H	-4.839922000	0.767377000	3.607033000
H	-4.907101000	-0.258926000	2.161561000
C	0.278958000	-3.710602000	2.054163000
F	0.625947000	-3.268589000	3.295440000
F	-1.058497000	-3.971431000	2.082129000
F	0.918484000	-4.889466000	1.849965000
C	0.400961000	3.754471000	-2.121887000
F	1.063252000	4.917245000	-1.899299000
F	0.765444000	3.307892000	-3.355801000
F	-0.929530000	4.048040000	-2.179101000
C	-3.358967000	-1.517822000	-2.073899000
F	-3.058060000	-1.083416000	-3.330568000
F	-3.072093000	-2.850252000	-2.034625000
F	-4.699445000	-1.386643000	-1.908925000

12) $[Ru((C_6H_5)COCHCO(C_6H_5))_3]$ complex 7

Ru	0.026428000	-0.255569000	-0.057275000
O	-1.000410000	-1.726437000	-0.983188000
O	-1.266363000	-0.202407000	1.463010000
O	1.329521000	-0.235742000	-1.581367000
O	-1.103933000	1.050135000	-1.061525000
O	1.088630000	1.146981000	0.886657000
O	1.122991000	-1.627645000	0.943034000
C	-2.222169000	-2.033311000	-0.713760000
C	-2.457564000	-0.724503000	1.419717000
C	2.556811000	-0.658519000	-1.488550000
C	-1.032841000	2.335193000	-0.889991000
C	0.850996000	2.415671000	0.785175000
C	2.368552000	-1.857710000	0.711849000
C	-2.941685000	-1.551147000	0.400533000
C	3.079348000	-1.379774000	-0.410223000
C	-0.144075000	2.991347000	-0.024532000
H	-3.958654000	-1.908281000	0.514161000
H	-0.263902000	4.066704000	0.052876000
H	4.137656000	-1.610043000	-0.447331000

C	3.417388000	-0.310739000	-2.650816000
C	4.591081000	-1.020581000	-2.957383000
C	3.043479000	0.762153000	-3.477814000
C	5.372767000	-0.656849000	-4.050439000
C	3.829711000	1.127394000	-4.566254000
C	4.998443000	0.419364000	-4.856818000
H	2.133555000	1.312023000	-3.247443000
H	6.275111000	-1.222290000	-4.278831000
H	3.531375000	1.968128000	-5.190497000
H	5.612080000	0.702219000	-5.710542000
C	-2.852497000	-3.004056000	-1.647587000
C	-2.028411000	-3.814545000	-2.449563000
C	-4.246381000	-3.126865000	-1.773421000
C	-2.583360000	-4.730634000	-3.337101000
C	-4.798691000	-4.036247000	-2.672071000
C	-3.970450000	-4.845417000	-3.453418000
H	-0.948438000	-3.720462000	-2.357890000
H	-4.911128000	-2.492699000	-1.188853000
H	-1.930684000	-5.359988000	-3.942957000
H	-5.881089000	-4.110344000	-2.765584000
H	-4.404389000	-5.559250000	-4.151580000
C	-2.012001000	3.121016000	-1.687763000
C	-1.774888000	4.455693000	-2.055711000
C	-3.207131000	2.508933000	-2.101127000
C	-2.711390000	5.158743000	-2.808759000
C	-4.145609000	3.217105000	-2.845373000
C	-3.901401000	4.544867000	-3.202663000
H	-0.842545000	4.943290000	-1.778300000
H	-3.394830000	1.474812000	-1.819323000
H	-2.506933000	6.189281000	-3.095428000
H	-5.072858000	2.732260000	-3.147029000
H	-4.633439000	5.097741000	-3.789187000
H	4.887390000	-1.877895000	-2.357119000
C	3.052665000	-2.697073000	1.729951000
C	4.228120000	-3.415117000	1.449859000
C	2.501833000	-2.779579000	3.021020000
C	4.837182000	-4.185708000	2.436462000
C	3.116087000	-3.545253000	4.006311000
C	4.286819000	-4.251422000	3.718162000
H	4.659315000	-3.396467000	0.450886000
H	1.592072000	-2.224302000	3.240714000
H	5.742935000	-4.742828000	2.201729000
H	2.683532000	-3.590017000	5.004976000
H	4.766949000	-4.852015000	4.488846000
C	-3.322358000	-0.373563000	2.577400000
C	-4.392346000	-1.183840000	2.989878000
C	-3.059322000	0.812068000	3.286819000
C	-5.181594000	-0.814342000	4.075728000
C	-3.856804000	1.184291000	4.364597000
C	-4.920544000	0.373175000	4.763973000
H	-4.597504000	-2.123191000	2.479968000

H	-2.229614000	1.440939000	2.972172000
H	-6.000096000	-1.459467000	4.390755000
H	-3.648184000	2.112301000	4.895627000
H	-5.541209000	0.661763000	5.610670000
C	1.719651000	3.279465000	1.635435000
C	2.394480000	2.702671000	2.725761000
C	1.903541000	4.648856000	1.378252000
C	3.214243000	3.475779000	3.544122000
C	2.729917000	5.418858000	2.194683000
C	3.385252000	4.837164000	3.282226000
H	2.259818000	1.641023000	2.924031000
H	1.420978000	5.119229000	0.522481000
H	3.722405000	3.014343000	4.390992000
H	2.867700000	6.477142000	1.975791000
H	4.029652000	5.442310000	3.920589000

13) [Ru($CH_3COCHCO(C_6H_5))_3$] *fac* complex 8

Ru	0.198692000	-0.176345000	1.010718000
O	1.368514000	0.844395000	-0.249418000
O	1.776985000	-0.735972000	2.151477000
O	-1.354766000	0.315796000	-0.153315000
O	0.409308000	-1.834053000	-0.132859000
O	-1.023597000	-1.122151000	2.289997000
O	0.050124000	1.442209000	2.181460000
C	2.575182000	1.231110000	0.035846000
C	2.927472000	-0.165940000	2.096222000
C	-2.038100000	1.405951000	-0.006426000
C	-0.363060000	-2.860063000	-0.072984000
C	-1.598631000	-2.257758000	2.030420000
C	-0.852635000	2.352635000	2.011170000
C	3.321371000	0.785571000	1.133787000
C	-1.830330000	2.362589000	1.003791000
C	-1.339720000	-3.071488000	0.926963000
H	4.343120000	1.142214000	1.215583000
H	-1.889582000	-4.006971000	0.892253000
H	-2.520075000	3.200358000	1.030197000
C	-3.126666000	1.596645000	-1.002222000
C	-3.632591000	2.867868000	-1.321382000
C	-3.655742000	0.474692000	-1.664036000
C	-4.640320000	3.011027000	-2.271860000
C	-4.668994000	0.620039000	-2.606932000
C	-5.165366000	1.888438000	-2.915636000
H	-3.266996000	-0.511539000	-1.420532000
H	-5.012304000	4.004797000	-2.516051000
H	-5.075431000	-0.260398000	-3.103899000
H	-5.954915000	2.002193000	-3.656851000
C	3.163665000	2.188941000	-0.937935000
C	2.619448000	2.272855000	-2.231085000
C	4.229743000	3.037879000	-0.597288000
C	3.141131000	3.166341000	-3.162847000

C	4.741689000	3.939133000	-1.528152000
C	4.203104000	4.003381000	-2.815133000
H	1.789088000	1.622386000	-2.495130000
H	4.648026000	3.018610000	0.407261000
H	2.716421000	3.211047000	-4.164848000
H	5.560118000	4.598751000	-1.244220000
H	4.607285000	4.706494000	-3.542009000
C	-0.130655000	-3.890526000	-1.120724000
C	-1.102798000	-4.846977000	-1.460167000
C	1.094127000	-3.899598000	-1.809057000
C	-0.851864000	-5.786219000	-2.457050000
C	1.346376000	-4.846916000	-2.795681000
C	0.373764000	-5.794767000	-3.123952000
H	-2.071606000	-4.847565000	-0.964398000
H	1.846904000	-3.156633000	-1.551749000
H	-1.620086000	-6.514024000	-2.716214000
H	2.305923000	-4.847808000	-3.311814000
H	0.569385000	-6.534820000	-3.899058000
H	-3.220074000	3.756770000	-0.846954000
C	3.909639000	-0.609885000	3.148298000
H	4.874617000	-0.106560000	3.046347000
H	3.498011000	-0.402457000	4.144330000
H	4.060012000	-1.695089000	3.078494000
C	-0.799904000	3.478182000	3.010866000
H	0.159116000	4.005726000	2.919560000
H	-1.613680000	4.193281000	2.865491000
H	-0.851828000	3.072781000	4.029523000
C	-2.601322000	-2.690473000	3.066726000
H	-2.105860000	-2.789292000	4.041410000
H	-3.374359000	-1.918001000	3.173314000
H	-3.074503000	-3.640794000	2.807523000

14) [Ru(CH_3COCHCO(C_6H_5))$_3$] *mer* complex 8

Ru	3.319227000	4.123084000	2.198511000
O	4.747729000	4.232592000	0.804069000
O	2.124675000	5.463895000	1.315537000
O	4.469048000	2.764829000	3.165718000
O	4.260275000	5.589514000	3.190826000
O	1.903694000	3.958768000	3.634479000
O	2.372964000	2.694146000	1.153278000
C	4.682442000	5.013640000	-0.227535000
C	2.426158000	6.054871000	0.205478000
C	4.599116000	1.536453000	2.807205000
C	3.697215000	6.288373000	4.130117000
C	1.654309000	4.873128000	4.504404000
C	2.800045000	1.472724000	1.053410000
C	3.606302000	5.867649000	-0.530081000
C	3.848701000	0.912702000	1.785091000
C	2.470630000	6.004980000	4.731874000
H	3.675119000	6.431709000	-1.455518000

H	2.140517000	6.710093000	5.489212000
H	4.080511000	-0.125254000	1.565777000
C	0.416572000	4.662823000	5.306548000
C	-0.566978000	3.786441000	4.814704000
C	0.192199000	5.306599000	6.535360000
C	-1.745055000	3.569649000	5.524314000
C	-0.986198000	5.084151000	7.245921000
C	-1.959453000	4.218426000	6.742983000
H	-0.396599000	3.287760000	3.863028000
H	0.945226000	5.968926000	6.955839000
H	-2.500775000	2.895198000	5.124489000
H	-1.142136000	5.586007000	8.199757000
H	-2.881502000	4.049178000	7.298781000
C	5.613203000	0.761712000	3.570038000
C	5.551499000	-0.635873000	3.694360000
C	6.660824000	1.452171000	4.204551000
C	6.513248000	-1.322678000	4.430809000
C	7.625745000	0.762199000	4.931276000
C	7.555247000	-0.627954000	5.048503000
H	6.710577000	2.534722000	4.108958000
H	6.445079000	-2.405063000	4.528503000
H	8.437845000	1.309430000	5.407904000
H	8.308005000	-1.167494000	5.620737000
C	5.857561000	4.939398000	-1.136198000
C	6.667156000	3.791511000	-1.111939000
C	6.196258000	5.984801000	-2.011089000
C	7.772317000	3.685319000	-1.950512000
C	7.307691000	5.879052000	-2.842999000
C	8.097369000	4.728607000	-2.819596000
H	6.409599000	2.981166000	-0.433201000
H	5.606197000	6.899009000	-2.028456000
H	8.383297000	2.783985000	-1.927310000
H	7.561986000	6.702884000	-3.508066000
H	8.963938000	4.647004000	-3.473392000
H	4.734084000	-1.190781000	3.238921000
C	1.375717000	7.012017000	-0.294938000
H	1.671011000	7.491915000	-1.231431000
H	0.429827000	6.475532000	-0.448433000
H	1.190429000	7.785031000	0.462587000
C	2.038215000	0.632251000	0.066510000
H	2.070337000	1.105630000	-0.923647000
H	2.439463000	-0.382171000	-0.003938000
H	0.983394000	0.580312000	0.365775000
C	4.497532000	7.479833000	4.575776000
H	3.990571000	8.042623000	5.363598000
H	5.476742000	7.148066000	4.945536000
H	4.682631000	8.140992000	3.719218000

15) [Ru($CH_3COCHCOCH_3$)$_3$] complex 9

Ru	-0.126664000	-0.005460000	0.005802000

O	-1.558586000	-0.876561000	1.153088000
O	-0.085634000	-1.557960000	-1.271004000
O	-0.106881000	1.546934000	1.282879000
O	1.234129000	-0.902933000	1.176934000
O	1.228451000	0.906452000	-1.160801000
O	-1.562631000	0.851072000	-1.147743000
C	-1.885704000	-2.114308000	1.070211000
C	-0.618220000	-2.713497000	-1.016832000
C	-0.650196000	2.697096000	1.026659000
C	2.507943000	-0.762559000	1.008403000
C	2.503036000	0.781639000	-0.986649000
C	-1.899522000	2.086378000	-1.068000000
C	-2.866734000	-2.588499000	2.110723000
C	-1.432761000	-3.011037000	0.078795000
C	-0.320404000	-3.772502000	-2.041342000
C	-0.371051000	3.757858000	2.054254000
C	-1.460530000	2.987275000	-0.074072000
C	3.355766000	-1.521620000	1.993358000
C	3.122257000	0.013803000	0.012746000
C	3.346467000	1.548280000	-1.969731000
C	-2.878573000	2.552177000	-2.113984000
H	-3.817251000	-2.051132000	1.989656000
H	-3.054556000	-3.662697000	2.035907000
H	-2.485677000	-2.353639000	3.112376000
H	-1.813447000	-4.027557000	0.137596000
H	0.766598000	-3.905841000	-2.127608000
H	-0.782746000	-4.729175000	-1.786183000
H	-0.685356000	-3.447716000	-3.024589000
H	3.096214000	-1.218711000	3.016193000
H	3.145876000	-2.595567000	1.910008000
H	4.424194000	-1.352782000	1.829058000
H	4.209737000	0.020545000	0.014909000
H	3.130717000	2.621356000	-1.883011000
H	4.415865000	1.385617000	-1.805681000
H	3.088693000	1.247023000	-2.993516000
H	0.713564000	3.904954000	2.146782000
H	-0.844065000	4.709092000	1.798313000
H	-0.737055000	3.426724000	3.035116000
H	-1.850427000	4.000172000	-0.134918000
H	-3.830313000	2.017602000	-1.988897000
H	-3.064927000	3.627169000	-2.048649000
H	-2.497307000	2.307673000	-3.112892000

16) [Ru(C(CH$_3$)$_3$COCHCOC(CH$_3$)$_3$)$_3$] complex 10

Ru	-0.024554000	0.153156000	0.033529000
O	1.446262000	-0.572350000	-1.121472000
O	-0.106879000	-1.465870000	1.211487000
O	-0.024619000	1.809683000	-1.135883000
O	-1.327752000	-0.713946000	-1.221333000
O	-1.515402000	0.966272000	1.139897000

O	1.329912000	0.947044000	1.286461000
C	1.932165000	-1.756526000	-0.990862000
C	0.602449000	-2.523914000	1.015114000
C	0.847480000	2.741681000	-1.068011000
C	-2.590073000	-0.849988000	-0.979076000
C	-2.746493000	0.631087000	1.050342000
C	2.034816000	1.996625000	1.022508000
C	1.553634000	-2.683799000	-0.004078000
C	1.848100000	2.838342000	-0.077203000
C	-3.273450000	-0.250105000	0.082304000
H	2.064280000	-3.642992000	-0.032634000
H	-4.341983000	-0.437655000	0.134244000
H	2.512582000	3.694281000	-0.147147000
C	3.008276000	-2.151896000	-2.025444000
C	4.321189000	-2.507092000	-1.296760000
C	3.273159000	-0.991645000	-2.994619000
H	2.367372000	-0.717684000	-3.546579000
H	3.622262000	-0.098148000	-2.464981000
H	4.043483000	-1.293566000	-3.717222000
H	4.688213000	-1.655501000	-0.710191000
H	5.091404000	-2.771017000	-2.034693000
H	4.196854000	-3.359790000	-0.619887000
C	2.517259000	-3.373583000	-2.829878000
H	1.576999000	-3.149231000	-3.349221000
H	3.268934000	-3.643739000	-3.584555000
H	2.352903000	-4.247207000	-2.188831000
C	-3.365578000	-1.730805000	-1.981085000
C	-2.419221000	-2.296314000	-3.050140000
C	-4.458709000	-0.891312000	-2.672718000
H	-5.188665000	-0.497959000	-1.955222000
H	-4.018974000	-0.043227000	-3.213419000
H	-4.998821000	-1.515059000	-3.397284000
H	-1.945495000	-1.496123000	-3.629122000
H	-2.992467000	-2.931328000	-3.738356000
H	-1.622598000	-2.899653000	-2.601439000
C	-4.020720000	-2.906139000	-1.222227000
H	-3.264051000	-3.517929000	-0.715871000
H	-4.738699000	-2.558382000	-0.469271000
H	-4.558678000	-3.547112000	-1.933118000
C	0.749888000	3.836579000	-2.150814000
C	2.068546000	3.871060000	-2.951533000
C	0.512209000	5.206136000	-1.482201000
H	-0.417786000	5.202127000	-0.899108000
H	1.334149000	5.484788000	-0.813086000
H	0.426909000	5.982052000	-2.254765000
H	2.926885000	4.119899000	-2.317511000
H	1.998276000	4.631694000	-3.740631000
H	2.264323000	2.901724000	-3.427033000
C	-0.410572000	3.539635000	-3.109837000
H	-1.369572000	3.508935000	-2.579841000
H	-0.459004000	4.326732000	-3.873923000

H	-0.277235000	2.574790000	-3.612339000
C	3.138719000	2.323030000	2.049494000
C	2.826931000	3.677775000	2.718757000
C	4.501714000	2.400712000	1.332209000
H	4.735117000	1.454497000	0.827784000
H	4.527879000	3.201462000	0.583514000
H	5.292362000	2.600264000	2.067975000
H	2.807945000	4.496471000	1.989699000
H	3.597574000	3.906551000	3.467591000
H	1.854365000	3.649543000	3.227176000
C	3.208518000	1.233981000	3.131897000
H	3.425937000	0.252078000	2.696701000
H	4.005546000	1.484558000	3.844001000
H	2.263708000	1.153658000	3.680661000
C	0.353809000	-3.680924000	2.004406000
C	-0.742596000	-3.302362000	3.011063000
C	1.658256000	-3.987206000	2.772277000
H	2.461435000	-4.308725000	2.099014000
H	2.005856000	-3.104012000	3.322691000
H	1.479293000	-4.793524000	3.496194000
H	-0.461055000	-2.421044000	3.597369000
H	-0.905177000	-4.141604000	3.699100000
H	-1.688335000	-3.076920000	2.505428000
C	-0.094848000	-4.936531000	1.228882000
H	-1.017319000	-4.742665000	0.665822000
H	0.670351000	-5.278870000	0.521583000
H	-0.292073000	-5.754055000	1.935088000
C	-3.700542000	1.272151000	2.077642000
C	-4.798386000	2.070307000	1.344524000
C	-2.930537000	2.220265000	3.007574000
H	-2.139000000	1.692816000	3.551000000
H	-2.461436000	3.035934000	2.445863000
H	-3.626016000	2.653934000	3.737042000
H	-4.361323000	2.863812000	0.725762000
H	-5.465094000	2.539022000	2.079888000
H	-5.409530000	1.429361000	0.696365000
C	-4.350161000	0.154260000	2.920659000
H	-3.588089000	-0.439119000	3.440885000
H	-5.011916000	0.600438000	3.674422000
H	-4.950659000	-0.526010000	2.304081000

17) [Ru(EtCOCHCOEt)$_3$] complex 11

Ru	-0.030726000	-0.217853000	-0.203200000
O	1.210744000	0.897095000	0.908127000
O	-0.746683000	1.330042000	-1.265582000
O	0.648877000	-1.815269000	0.844698000
O	-1.437752000	0.107656000	1.189412000
O	-1.265524000	-1.405835000	-1.297087000
O	1.404514000	-0.423362000	-1.597221000
C	1.288895000	2.179503000	0.800354000

C	-0.379275000	2.551468000	-1.057803000
C	1.831474000	-2.292227000	0.711063000
C	-2.696991000	-0.130770000	1.003888000
C	-2.536831000	-1.446811000	-1.133651000
C	2.491813000	-1.103858000	-1.402533000
C	2.257704000	2.833381000	1.760787000
C	0.560170000	2.974155000	-0.102180000
C	-1.068217000	3.574636000	-1.931486000
C	2.242081000	-3.295626000	1.766185000
C	2.732543000	-1.956248000	-0.322520000
C	-3.605320000	0.419982000	2.080627000
C	-3.241416000	-0.838120000	-0.072478000
C	-3.302811000	-2.230828000	-2.177166000
C	3.545638000	-0.911671000	-2.466625000
H	3.239959000	2.360682000	1.615307000
H	2.362933000	3.895456000	1.510370000
H	0.750974000	4.044041000	-0.060459000
H	-0.551877000	4.538078000	-1.844733000
H	-0.978558000	3.242220000	-2.975262000
H	-3.522021000	1.517315000	2.040572000
H	-4.645015000	0.165215000	1.844865000
H	-4.319877000	-0.975479000	-0.066335000
H	-3.265653000	-1.646422000	-3.110663000
H	-4.356431000	-2.292247000	-1.882531000
H	1.464318000	-4.071828000	1.814420000
H	3.178486000	-3.781439000	1.470271000
H	3.694074000	-2.465168000	-0.310305000
H	4.331700000	-1.666132000	-2.348549000
H	3.070893000	-1.074446000	-3.444454000
C	1.830142000	2.669307000	3.225501000
H	2.581645000	3.104994000	3.893976000
H	0.873417000	3.172239000	3.414610000
H	1.715551000	1.608779000	3.476737000
C	-2.553726000	3.735585000	-1.576132000
H	-2.674330000	4.104000000	-0.549852000
H	-3.032052000	4.452541000	-2.253334000
H	-3.078633000	2.776852000	-1.660557000
C	4.151403000	0.501262000	-2.424525000
H	4.880666000	0.626121000	-3.233278000
H	3.371638000	1.262436000	-2.542289000
H	4.664466000	0.680630000	-1.471270000
C	2.391880000	-2.637468000	3.145739000
H	3.183421000	-1.876842000	3.135334000
H	1.456762000	-2.151298000	3.445466000
H	2.650287000	-3.389293000	3.901280000
C	-2.729722000	-3.627541000	-2.433160000
H	-1.677006000	-3.568752000	-2.728844000
H	-2.797738000	-4.250729000	-1.532693000
H	-3.286723000	-4.125369000	-3.234954000
C	-3.238921000	-0.058757000	3.491831000
H	-2.202922000	0.203381000	3.732792000

H	-3.896429000	0.408911000	4.233372000
H	-3.347343000	-1.147285000	3.578394000

18) [Ru(PrCOCHCOPr)$_3$] complex 12

Ru	-0.103047000	-0.180905000	-0.354804000
O	1.354428000	0.646850000	0.745787000
O	-0.689513000	1.547217000	-1.194336000
O	0.439844000	-1.954349000	0.464653000
O	-1.343309000	0.129197000	1.192436000
O	-1.554353000	-1.085349000	-1.455127000
O	1.189313000	-0.404110000	-1.879861000
C	1.621387000	1.908272000	0.721634000
C	-0.115833000	2.677692000	-0.938448000
C	1.544279000	-2.546046000	0.191786000
C	-2.628344000	-0.009798000	1.127721000
C	-2.802239000	-1.043858000	-1.159008000
C	2.204764000	-1.212240000	-1.833290000
C	2.735990000	2.334103000	1.648607000
C	0.958341000	2.872056000	-0.056460000
C	-0.716834000	3.868618000	-1.649055000
C	1.892269000	-3.709215000	1.089076000
C	2.413530000	-2.195582000	-0.864342000
C	-3.362335000	0.417714000	2.376259000
C	-3.338812000	-0.530862000	0.040916000
C	-3.726890000	-1.645830000	-2.191229000
C	3.210106000	-1.019925000	-2.940903000
H	3.593161000	1.665300000	1.481995000
H	3.054639000	3.354738000	1.400955000
H	1.320258000	3.893770000	0.036473000
H	0.011207000	4.689941000	-1.672739000
H	-0.930973000	3.578371000	-2.687755000
H	-3.092253000	1.466066000	2.579408000
H	-4.444631000	0.381814000	2.200767000
H	-4.419344000	-0.587279000	0.149956000
H	-3.527369000	-1.147431000	-3.152187000
H	-4.769694000	-1.437383000	-1.920807000
H	1.034009000	-4.397130000	1.107584000
H	2.747285000	-4.256616000	0.673224000
H	3.306962000	-2.805266000	-0.977441000
H	3.900539000	-1.871757000	-2.968669000
H	2.669141000	-0.995021000	-3.898962000
C	2.327583000	2.261096000	3.130626000
H	1.461124000	2.917338000	3.296635000
H	1.996268000	1.237877000	3.353034000
C	-2.017439000	4.352863000	-0.983136000
H	-2.730937000	3.518189000	-0.950085000
H	-1.804609000	4.628975000	0.059885000
C	4.004661000	0.291400000	-2.780349000
H	4.530966000	0.274589000	-1.814426000
H	3.297337000	1.130266000	-2.739597000

C	2.204454000	-3.261095000	2.528876000
H	3.043359000	-2.550734000	2.510129000
H	1.338064000	-2.712223000	2.921592000
C	-3.522058000	-3.162007000	-2.367324000
H	-2.463489000	-3.350369000	-2.593220000
H	-3.737034000	-3.666586000	-1.414213000
C	-2.997672000	-0.432633000	3.604924000
H	-1.908086000	-0.407912000	3.736341000
H	-3.269983000	-1.480285000	3.409478000
C	5.006722000	0.501579000	-3.912682000
H	4.500721000	0.543913000	-4.886809000
H	5.559949000	1.440432000	-3.779901000
H	5.738931000	-0.316236000	-3.951429000
C	2.541948000	-4.439283000	3.439793000
H	3.420916000	-4.985932000	3.072952000
H	2.760942000	-4.099246000	4.459563000
H	1.706033000	-5.149302000	3.494014000
C	-3.692550000	0.056106000	4.873782000
H	-3.412305000	1.093387000	5.102506000
H	-4.785072000	0.021663000	4.767705000
H	-3.420270000	-0.563392000	5.736940000
C	-4.405919000	-3.737665000	-3.471482000
H	-5.470696000	-3.574012000	-3.254768000
H	-4.185912000	-3.265555000	-4.438211000
H	-4.248375000	-4.818539000	-3.580937000
C	3.467065000	2.661337000	4.065292000
H	3.154474000	2.605788000	5.115343000
H	4.334269000	2.000035000	3.937040000
H	3.799318000	3.689445000	3.868407000
C	-2.634519000	5.542510000	-1.718408000
H	-2.880350000	5.281186000	-2.756474000
H	-3.557329000	5.874933000	-1.227137000
H	-1.941537000	6.393903000	-1.743285000

19) [Ru(BuCOCHCOBu)$_3$] complex 13

Ru	-0.079576000	0.199265000	-0.478228000
O	1.453957000	-0.189184000	0.750141000
O	0.019394000	2.190747000	-0.248988000
O	-0.218155000	-1.814644000	-0.730248000
O	-1.286016000	0.064735000	1.116328000
O	-1.636155000	0.520462000	-1.742472000
O	1.198719000	0.382353000	-2.026383000
C	2.072988000	0.725487000	1.413848000
C	0.860095000	2.762574000	0.550922000
C	0.651349000	-2.500146000	-1.375934000
C	-2.521440000	0.453420000	1.118383000
C	-2.813305000	0.849207000	-1.347998000
C	1.866856000	-0.617398000	-2.515705000
C	3.149103000	0.206460000	2.339479000
C	1.814411000	2.106417000	1.342865000

C	0.745209000	4.268088000	0.606922000
C	0.564566000	-3.996563000	-1.197047000
C	1.658159000	-1.962813000	-2.207531000
C	-3.178134000	0.438195000	2.478423000
C	-3.252337000	0.846465000	-0.006826000
C	-3.785817000	1.210322000	-2.445746000
C	2.957453000	-0.229761000	-3.484043000
H	3.817686000	-0.431991000	1.742549000
H	3.743599000	1.042673000	2.727879000
H	2.442182000	2.744373000	1.964116000
H	1.603104000	4.685456000	1.149114000
H	0.787221000	4.649761000	-0.425032000
H	-2.587756000	1.095024000	3.138327000
H	-4.186432000	0.864443000	2.405495000
H	-4.286289000	1.137957000	0.163517000
H	-3.271655000	1.885411000	-3.144862000
H	-4.636001000	1.756358000	-2.016787000
H	-0.495630000	-4.279131000	-1.180426000
H	1.040252000	-4.503073000	-2.048395000
H	2.300921000	-2.680526000	-2.711824000
H	3.280429000	-1.111042000	-4.051552000
H	2.543811000	0.497376000	-4.197864000
C	2.565519000	-0.609978000	3.506929000
H	1.904736000	0.043128000	4.097101000
H	1.933976000	-1.409350000	3.094432000
C	-0.566473000	4.735939000	1.257976000
H	-1.411666000	4.274390000	0.726067000
H	-0.608092000	4.367299000	2.295064000
C	4.166788000	0.400820000	-2.766261000
H	4.569268000	-0.320286000	-2.037949000
H	3.826132000	1.273130000	-2.190433000
C	1.248358000	-4.438342000	0.112666000
H	2.283988000	-4.067509000	0.111548000
H	0.741687000	-3.948683000	0.958737000
C	-4.297869000	-0.016713000	-3.219653000
H	-3.437083000	-0.573805000	-3.617558000
H	-4.819818000	-0.692635000	-2.525252000
C	-3.239379000	-0.963693000	3.104883000
H	-2.223553000	-1.383141000	3.135350000
H	-3.836615000	-1.625187000	2.456467000
C	5.274209000	0.818530000	-3.735046000
H	5.596473000	-0.058706000	-4.316089000
H	4.865828000	1.537326000	-4.462078000
C	1.258869000	-5.956580000	0.318793000
H	1.866289000	-6.176388000	1.209188000
H	1.776491000	-6.432312000	-0.529843000
C	-3.837877000	-0.954180000	4.513286000
H	-3.241306000	-0.283507000	5.152604000
H	-4.850428000	-0.523172000	4.475703000
C	-5.237644000	0.366947000	-4.363142000
H	-6.084737000	0.940851000	-3.957407000

H	-4.708020000	1.043988000	-5.050788000
C	3.630489000	-1.216254000	4.427692000
H	3.122169000	-1.647026000	5.303545000
H	4.277823000	-0.414158000	4.814245000
C	-0.719842000	6.257046000	1.250774000
H	-0.665406000	6.618976000	0.212294000
H	0.133141000	6.711013000	1.779643000
C	-3.895854000	-2.345353000	5.143430000
H	-2.892653000	-2.786257000	5.219801000
H	-4.323866000	-2.309925000	6.153439000
H	-4.514133000	-3.026450000	4.541484000
C	-5.762031000	-0.842347000	-5.135777000
H	-6.429787000	-0.536082000	-5.950731000
H	-4.936882000	-1.417597000	-5.576785000
H	-6.325303000	-1.518202000	-4.477468000
C	-0.124924000	-6.582485000	0.491107000
H	-0.662434000	-6.123186000	1.332922000
H	-0.744957000	-6.461098000	-0.406342000
H	-0.046598000	-7.658332000	0.692033000
C	6.481624000	1.434750000	-3.025876000
H	6.193901000	2.333100000	-2.463725000
H	6.926157000	0.724982000	-2.315331000
H	7.261214000	1.723617000	-3.742916000
C	-2.026598000	6.724941000	1.889184000
H	-2.090912000	6.404981000	2.937958000
H	-2.114031000	7.818330000	1.867005000
H	-2.895109000	6.308565000	1.360394000
C	4.488971000	-2.297013000	3.769365000
H	5.080919000	-1.900410000	2.934945000
H	5.191446000	-2.733970000	4.489930000
H	3.862546000	-3.109829000	3.377000000

20) [Ru(*i*PrCOCHCO*i*Pr)$_3$] complex 14

Ru	-0.000025000	0.023426000	0.000179000
O	-1.003159000	-1.337089000	1.078634000
O	1.002952000	-1.337321000	-1.078119000
O	-0.943918000	1.466641000	1.069860000
O	1.444563000	0.002358000	1.398033000
O	0.944198000	1.466253000	-1.069734000
O	-1.444608000	0.002403000	-1.397619000
C	-0.864228000	-2.608314000	0.916690000
C	0.863730000	-2.608508000	-0.916210000
C	-2.149163000	1.842562000	0.866078000
C	2.592980000	0.581559000	1.260710000
C	2.149642000	1.841679000	-0.866183000
C	-2.592898000	0.581907000	-1.260415000
C	-1.742936000	-3.465534000	1.821166000
C	-0.000363000	-3.229080000	0.000205000
C	1.742040000	-3.465920000	-1.820894000
C	-2.683584000	2.883350000	1.843300000

C	-2.964720000	1.418368000	-0.204037000
C	3.573090000	0.326728000	2.396297000
C	2.965077000	1.417543000	0.204042000
C	2.684474000	2.881738000	-1.843957000
C	-3.573241000	0.326685000	-2.395711000
H	-1.498403000	-4.516740000	1.615512000
H	-0.000593000	-4.317618000	0.000110000
H	1.496671000	-4.517046000	-1.615834000
H	4.505014000	0.851464000	2.146317000
H	3.966964000	1.839805000	0.247477000
H	3.758929000	2.997430000	-1.643383000
H	-3.758140000	2.998819000	1.643112000
H	-3.966478000	1.840918000	-0.247597000
H	-4.505027000	0.851714000	-2.145830000
C	1.449007000	-3.170587000	-3.298811000
H	1.669172000	-2.120756000	-3.528533000
H	2.073475000	-3.805869000	-3.942023000
H	0.396838000	-3.362357000	-3.544707000
C	3.226014000	-3.236536000	-1.493802000
H	3.448929000	-3.485335000	-0.448650000
H	3.856730000	-3.864911000	-2.137795000
H	3.494850000	-2.186271000	-1.661418000
C	-1.449198000	-3.171177000	3.299140000
H	-2.074183000	-3.806082000	3.942223000
H	-0.397170000	-3.364192000	3.544666000
H	-1.668156000	-2.121214000	3.529410000
C	-3.226848000	-3.234890000	1.494660000
H	-3.450270000	-3.483020000	0.449459000
H	-3.857817000	-3.863114000	2.138548000
H	-3.494855000	-2.184506000	1.662871000
C	-2.494412000	2.428552000	3.294936000
H	-3.010002000	1.479239000	3.487442000
H	-1.428804000	2.289428000	3.515748000
H	-2.896817000	3.183105000	3.982161000
C	-1.994855000	4.235401000	1.588465000
H	-2.390549000	4.995667000	2.273674000
H	-0.913243000	4.148444000	1.752752000
H	-2.159450000	4.581364000	0.560786000
C	-3.874984000	-1.173626000	-2.527864000
H	-4.590698000	-1.342569000	-3.342179000
H	-2.956170000	-1.729964000	-2.750966000
H	-4.305394000	-1.578401000	-1.603260000
C	-3.028593000	0.900603000	-3.712449000
H	-2.836508000	1.977791000	-3.630373000
H	-2.089565000	0.404498000	-3.985363000
H	-3.753508000	0.742524000	-4.520726000
C	3.874530000	-1.173578000	2.529318000
H	4.305142000	-1.578930000	1.605056000
H	4.589974000	-1.342206000	3.343933000
H	2.955548000	-1.729620000	2.752448000
C	3.028304000	0.901421000	3.712642000

H	2.089114000	0.405657000	3.985616000
H	3.753026000	0.743597000	4.521139000
H	2.836464000	1.978618000	3.629990000
C	1.995531000	4.233953000	-1.590629000
H	0.913995000	4.146762000	-1.755297000
H	2.159654000	4.580842000	-0.563187000
H	2.391465000	4.993641000	-2.276345000
C	2.495976000	2.425675000	-3.295319000
H	2.898714000	3.179638000	-3.983001000
H	3.011644000	1.476183000	-3.486780000
H	1.430463000	2.286380000	-3.516466000
